# Reasons that lead people to buy prescription medicines on the internet: a systematic review

**DOI:** 10.3389/fphar.2023.1239507

**Published:** 2023-08-31

**Authors:** Hamzeh Almomani, Amna Raza, Nilesh Patel, Parastou Donyai

**Affiliations:** ^1^ School of Pharmacy, University of Reading, Reading, United Kingdom; ^2^ Department of Pharmacy and Forensic Science, Faculty of Life Science and Medicine, King’s College London, London, United Kingdom

**Keywords:** prescription medicines, online pharmacy, theoretical domains framework, COM-B model, narrative synthesis, a systematic review, internet

## Abstract

**Aim:** This systematic review explores the factors that could influence consumer’s decision of purchasing prescription medicines using the Internet.

**Methods:** Relevant databases were searched to retrieve studies published from 2012 to 2021. The studies selected for inclusion were those focused on the consumer’s perspective and the purchase of prescription medicines. A narrative synthesis was employed. The Capability Opportunity Motivation-Behaviour (COM-B) and the Theoretical Domains Framework (TDF) were employed as conceptual lenses that guided the analysis.

**Results:** Seventeen studies were included. These studies have adopted various methodologies: qualitative method (*n* = 4), quantitative method (*n* = 12), and mixed methods (*n* = 1). The studies were based in Europe (*n* = 8), North America (*n* = 3), Middle East (*n* = 4), and 2 studies were conducted in several countries (multinational). The analysis of these studies revealed 7 themes that represent the reasons that lead people to buy prescription medicines via the Internet. These themes were the consumers’ beliefs about the outcomes of the purchase (perceived benefits and risks of the purchase), consumer’s emotions that could influence the purchasing decision, the factors that increase or decrease consumer’s level of behavioural control over the purchase (facilitators and barriers of the purchase), consumers knowledge about the purchase, the trusting beliefs that lead consumers to trust the online sellers of medicines, the social influencing factors, and the external environmental factors that could encourage the purchase.

**Discussion:** This study provides a comprehensive review of the breadth of reasons that drive people to buy prescription medicines via the Internet. Identifying those reasons could provide the basis for regulators to design evidence-based awareness campaigns to minimise the purchase of prescription medicines via the Internet. Furthermore, future research directions have been provided in this review to build upon the existing knowledge and address the research gaps in this area.

## 1 Introduction

Online sales of medicines have shown tremendous growth in recent years (Fittler et al., 2018a). Evidently, studies conducted in various countries (including the United States, Europe, and the Middle East) have found that the number of people purchasing medicines on the Internet has increased in recent years (Bowman et al., 2020; Alwhaibi et al., 2021; Jairoun et al., 2021; Moureaud et al., 2021; Fittler et al., 2022). One of these studies, conducted in Hungary, revealed a surprising tenfold increase in online medicine purchases from 2018 to 2020 (Fittler et al., 2022). People can purchase a wide range of medicines from the internet ranging from over-the-counter medicines (not needing a prescription) to medicines that are prescribed by an authorised prescriber (i.e., prescription medicines). Some consumers turn to the internet today to self-medicating and buy prescription medicines without having a valid prescription and without any input from healthcare providers. This is possible if consumers decided to purchase medicines from the illegal sellers of medicines operating on the internet.

The internet provides a platform that hosts both legal and illegal sellers of medicines. Legal online sellers of medicines are those who comply with safe pharmacy practices, and the laws and regulations of the country where the online seller operates (Mackey and Nayyar, 2016). Conversely, illegal online sellers of medicines are those who violate pharmacy regulations and laws.

A common practice of illegal sellers of medicines is offering the purchase of prescription medicines without any input from a healthcare provider, which includes not having a valid prescription (Mackey and Nayyar, 2016). Several studies have provided evidence of the availability of different types of prescription medicines on the internet and the easy accessibility to those medicines without the need for prescriptions (Boyd et al., 2017; Hockenhull et al., 2020; Whitfield et al., 2021). For example, researchers from Europe have explored the online availability of several active ingredients (including controlled medicines). They found that controlled medicines (such as tramadol) are widely available on the Internet and easily accessible to people without the need for a prescription (Fittler et al., 2013a). Likewise, a recent study conducted in the United States explored the online availability and accessibility of imatinib (anticancer medication) and found that those medicines are widely available online and could be obtained without any involvement of healthcare providers (Sun et al., 2021).

Laws regulating the sales of medicines over the Internet vary from one country to another. For example, in the United Kingdom, any legal sellers of medicines should be registered with the General Pharmaceutical Council (GPhC) and the Medicines and Healthcare products Regulatory Agency (MHRA) (GPC, 2022), while in the European Union (EU), licensed online pharmacies must be registered with the national competent authorities in the EU Member States (European Medicines Agency, 2019). Internationally, there are shortages of effective global regulations and laws that control and regulate online pharmacies (Fittler., 2013b; Gabay, 2015). Moreover, around two-thirds (66%) of countries worldwide do not have regulations that regulate the online trading of medicine (Hock et al., 2019). Thus, prescription medicines can be sold on the Internet by anyone in those countries (Hock et al., 2019). These shortages in international laws as well as the rapid development of technology have facilitated the expansion of illegal online pharmacies (Mackey and Nayyar, 2016). According to the Alliance for Safe Online Pharmacy (ASOP), over 95% of the 35,000 active online pharmacies worldwide operate illegally (ASOP, 2022). This uncontrolled proliferation of illegal online pharmacies may bring a lot of risks to people (e.g., risks of misusing the medicines as the healthcare provider is not involved in the purchase, or the risks of fake medicines which are widely available on the internet, and some of which may contain drugs of abuse or new psychoactive substances (NPS), which poses serious health risks to the consumers) and to the whole country (e.g., economic loss to the healthcare system due to utilizing ineffective treatments) (Fittler et al., 2013b).

The supply chain of prescription medicines over the Internet is complex and hard to track, which makes it impossible to keep the Internet free of illegal websites selling medicines (Fittler et al., 2013a; Lee et al., 2017). From another angle, one possible solution could come from focusing on the potential consumers themselves (Fittler et al., 2013a; Lee et al., 2017), as the consumers play a crucial role in the buying process of medicines from the Internet because they are the ones who ultimately make the decision to purchase medicines.

Illegal online sellers of medicines are exploiting rising consumers’ demand for convenience, lower cost, all day everyday accessibility, and the availability of different types of medications to sell various types of prescription medicines and fake medicines which are very profitable to online sellers, but potentially risky and life-threatening to people (Mackey and Nayyar, 2016; ASOP, 2022). Several public awareness campaigns have warned people about the danger of purchasing prescription medicines from the internet and about how to safely purchase medicines online (e.g., Fight the Fake, #FakeMeds), however, many people are still buying prescription medicines online without any input from a professional healthcare provider. According to a recent study, out of a sample size of 1,321 individuals, 136 (10.3%) have purchased prescription medicines online through online platforms without involving their doctors (Almomani et al., 2023a). Furthermore, according to an estimate by the United Kingdom government, 1 in 10 people bought fake medical products online in 2020 (MHRA, 2022). The reasons behind this behaviour are not well understood and need further investigation.

Previous systematic review studies in this field have explored the characteristics of the websites that sell medicines, the quality of pharmaceutical products purchased online, and the number of consumers who are purchasing medicines on the internet and their characteristics (Orizio et al., 2011; Long et al., 2022). However, none of these systematic reviews investigated the available evidence on the reasons that drive people to purchase prescription medicines via the Internet.

In light of the above, this systematic review aims to explore the prevalence of people purchasing prescription medicines from the Internet and to provide an overarching understanding of the reasons that could drive people to make this purchase. Additionally, this review provides directions for future research. To achieve the research objective, a narrative synthesis approach was undertaken. The Capability, Opportunity, Motivation- Behaviour model and the Theoretical Domains Framework were used to develop the coding of themes. To date, this is the first theory-based systematic review that focuses on the factors that could influence consumers’ decision of purchasing prescription medicines over the Internet.

## 2 Materials and methods

This systematic review is reported according to the Preferred Reporting Items for Systematic Reviews and Meta-Analyses (PRISMA) statement (Page et al., 2021).

### 2.1 Approach

The main aim of this study was to determine the breadth of reasons why people buy prescription medicines on the Internet. A narrative synthesis was employed because of the heterogenicity of methodologies between the included studies (Harden et al., 2004), as we included quantitative, qualitative, and mixed-methods studies. A narrative synthesis is an approach in which different types of studies (quantitative, qualitative, and mix-methods) were arranged into more homogenous groups (Barnett-Page and Thomas, 2009). In this type of synthesis, structured summaries are developed by comparing the similarities and differences between studies’ characteristics, context, quality, and findings (Harden et al., 2004; Barnett-Page and Thomas, 2009).

The current study employed the Capability, Opportunity, Motivation- Behaviour (COM-B) model and the Theoretical Domains Framework (TDF) as a conceptual lens through which the analysis was conducted.

**FIGURE 1:**
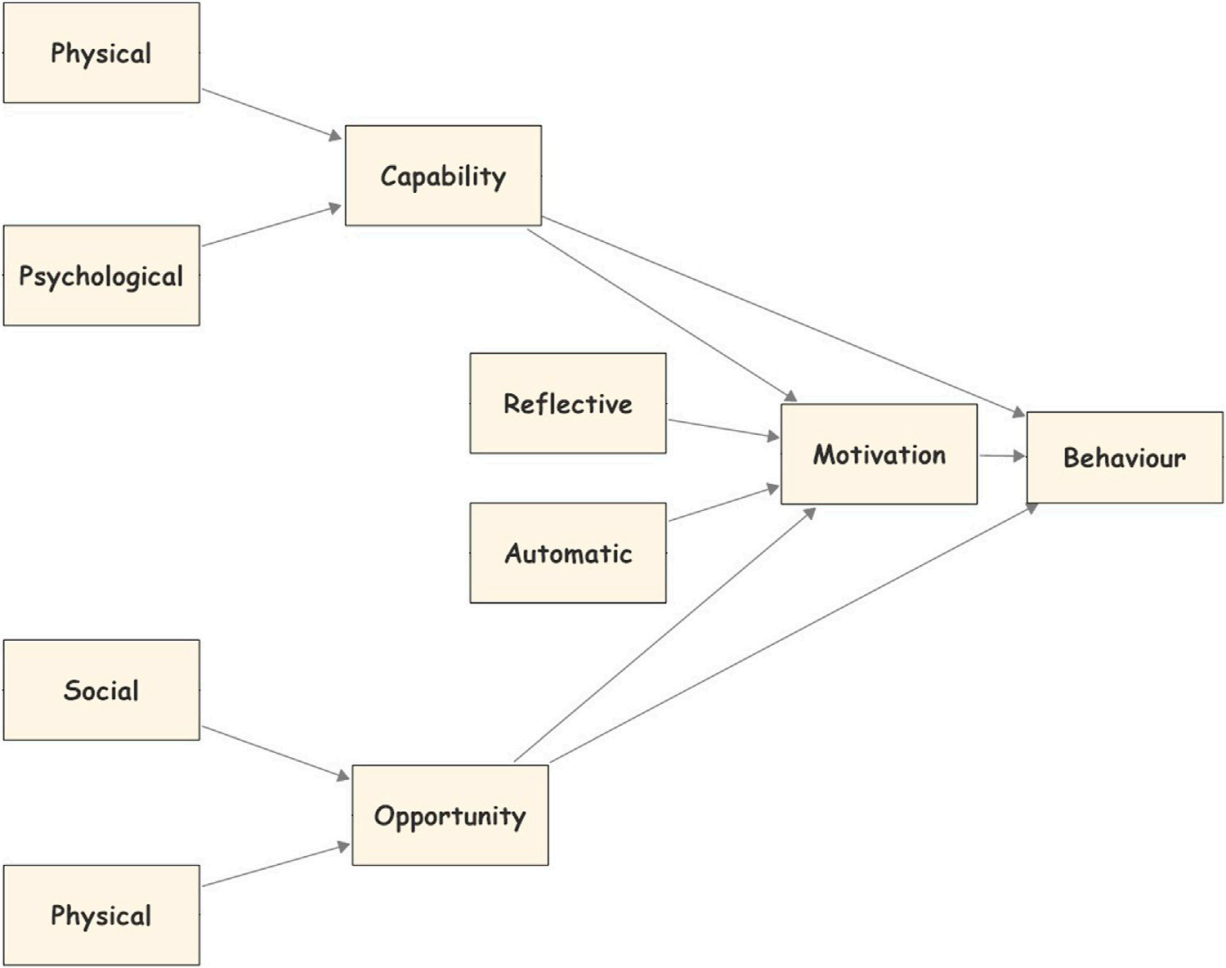
The COM-B model.

The COM-B model is one of the behavioural theories introduced by Michie *et al.* (2011). This model helps researchers to understand why people behave in a specific way. According to the COM-B model (Figure 1), a particular behaviour will occur only when an individual concerned has the capability (psychological or physical capability) and the opportunity (physical and social environment) to engage in the behaviour and is more motivated (reflective and automatic mechanisms) to enact that behaviour than any other behaviours (Michie et al., 2011). Capability is an attribute of the consumer that together with the opportunity facilitates the behaviour. It could be physical capability which represents the consumer’s body and physiques, or psychological capability which represent the consumer’s mental functioning (e.g., knowledge). Motivation is a sum of mental processes that energised consumers to make a specific behaviour. It could be reflective which represents the conscious thought processes (e.g., evaluations of the consequences), or automotive motivation which represents the affective processes (desires and emotions). Finally, the opportunity is an external environmental and social factor that together with capability facilitates the behaviour. It could be a physical opportunity which represents the external environmental circumstances, and the social opportunity which represents other people’s influence (social norms) (West and Michie, 2020).

The TDF is a theoretical framework synthesised from 33 behavioural theories and grouping the 128 constructs from these theories into domains (Michie et al., 2005; Cane et al., 2012). The TDF provided a comprehensive and theory-informed approach to identifying the predictors of behaviour (Atkins et al., 2017). Moreover, the TDF provides a theoretical lens through which to view the cognitive, affective (emotional), environmental and social influences on behaviour (Atkins et al., 2017). Appendix 1 illustrates the TDF domains and their definitions.

**FIGURE 2:**
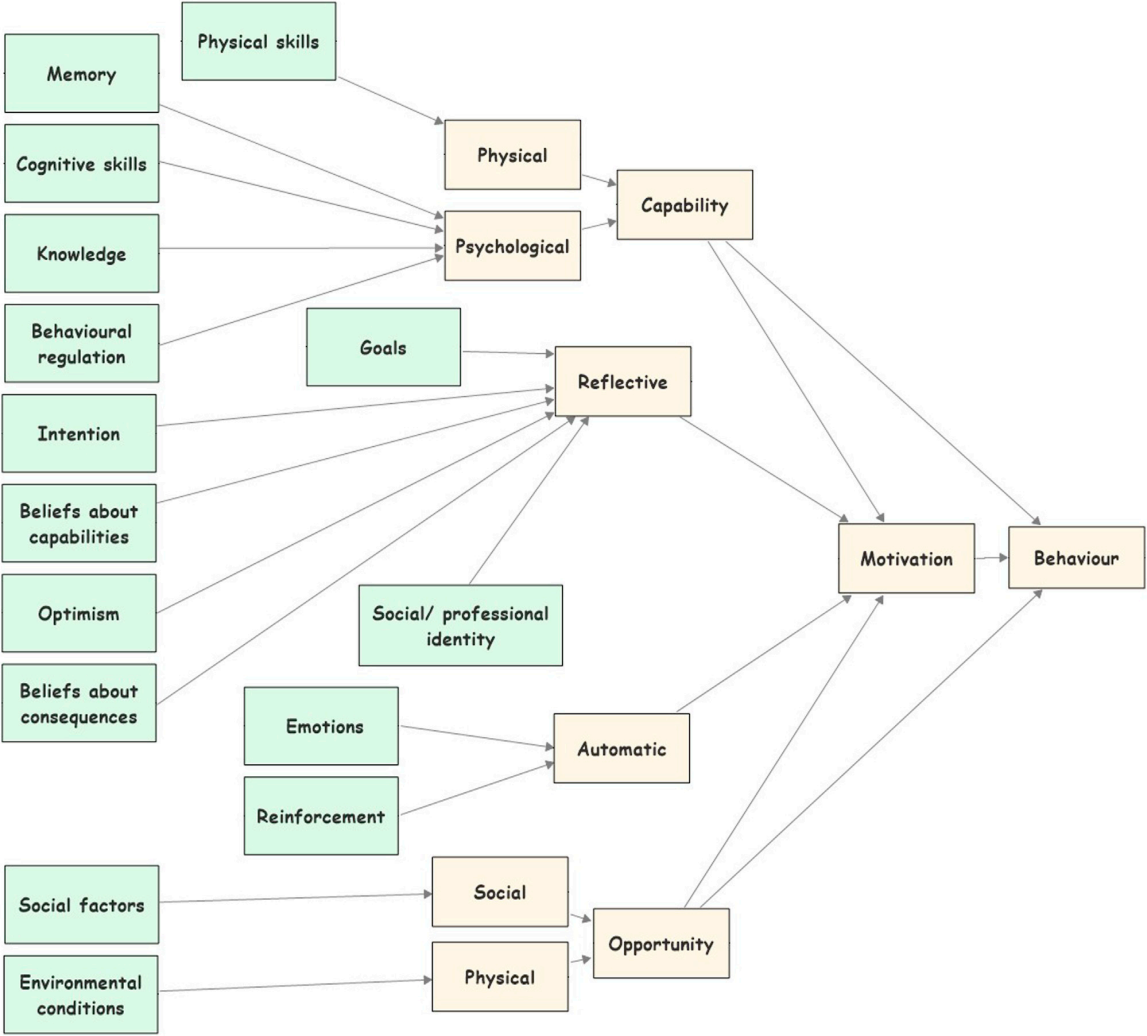
The TDF constructs mapped against the COM-B model.

The TDF constructs, in previous studies, were mapped against the COM-B model as shown in Figure 2 in order to unpack the COM-B model and to give a more detailed insight into the target behaviour (Zou et al., 2017; Alhusein et al., 2021; Timlin et al., 2021). Our study adopted the model shown in Figure 2 as a conceptual lens to analyse the previous studies’ findings to find the factors that could influence consumers’ decision to purchase prescription medicines from the Internet.

### 2.2 Review team

This study was conducted by four researchers (HM, PD, NP, AR). HM and AR are PhD researchers in the field of Pharmacy. Both PD and NP are experienced research-active academics.

### 2.3 Search strategy

The search was carried out using multiple relevant databases (i.e., Scopus, Web of Science, PubMed, and PsycINFO) to identify qualitative and quantitative published studies on the topic of ‘using the internet to purchase medicines requiring a prescription’. Database selection and the search strategy were conducted by HM and verified by PD and NP, in collaboration with the academic department’s librarian at the University.

The search was carried out using search terms related to the research topic (i.e., buying prescription medicines online). Search terms were developed by HA, PD, and NP.

### 2.4 Inclusion and exclusion criteria

The search was limited to articles published from the beginning of January 2012 to the end of December 2021. Studies were included if they were primary research articles (qualitative, quantitative, and mixed methods studies), published in the English language, focused on the consumer perspective, and focused on prescription medicines. Studies that focused on illegal drugs (e.g., marijuana, heroin, or cocaine) were excluded.

### 2.5 Study selection process

The selection process was conducted using the PRISMA Flow Diagram (Page et al., 2021). First, HM imported all records into the EndNote referencing management software to exclude any duplicates. Then, HM screened the titles to double-check and exclude any duplicates that EndNote may have missed. HM and AR then independently screened titles and abstracts before full-text screening to exclude any irrelevant studies using the inclusion/exclusion criteria. Additionally, the references and citations of the included articles were screened in order to retrieve articles that might have been missed in the database searches. Any inter-rater disagreement in the screening process was resolved through discussion. Each step in the selection process was verified by PD and NP.

### 2.6 Quality assessment

As the included articles used qualitative, quantitative, or mixed methods approaches, three methodological quality assessment tools were employed. The qualitative studies were assessed using the Critical Appraisal Skills Programme (CASP) qualitative studies checklist (CASP, 2018). The CASP qualitative checklist aims to assess various elements of qualitative research studies, including research aims, appropriate methodology, research design and strategy, methods of data collection and communication between researchers and participants, ethical considerations, the rigor of data analysis, and the clarity and value of study findings. The tool consists of a 10-point checklist that requires a *yes*, *no*, or *do not know.* The following guidelines were adopted: each item scored as one if the item received a rating of ‘*yes’*, zero if the item received a rating of ‘*no’* or ‘*do not know’* (Hafsteinsdóttir et al., 2017). Scores indicating high quality (≥8), medium quality (5–7), and low quality (≤4).

The quantitative studies were assessed using the Appraisal tool for Cross-Sectional Studies (AXIS) (Downes et al., 2016). The AXIS tool has been designed to assess the quality of observational cross-sectional studies. The components of the AXIS tool are based on a combination of evidence (systematic review of previous literature related to critical appraisal tools) and researchers’ opinions from a Delphi process. The tool consists of a 20-point checklist that requires a *yes*, *no*, or *do not know* (Yes = 1, No and Don’t know = 0). The following guidelines were adopted: Scores indicating high quality = 15–20, medium quality = 8–14, and low quality = 1–7 (Moor and Anderson, 2019).

The mixed methods studies were assessed using the Mixed Methods Appraisal Tool (MMAT) (Hong et al., 2018). The MMAT allows the appraisal of the methodological quality of empirical studies including mixed methods studies. Seventeen points were assessed that require a *yes*, *no*, or *do not know* (Yes = 1, No, and Cannot tell = 0). The following guidelines were adopted: Scores indicating high quality (90%–100%), medium quality (60%–89%), moderate-to-low quality (40%–59%); scores (≤39%) indicated a low quality (Dobersek et al., 2021).

Quality assessment checklists selection was done by HM, PD, and NP, in collaboration with the academic department’s librarian at the University. The quality assessment process was carried out by the HM, NP, and AR. HM assessed all the articles (qualitative, quantitative, and mixed methods studies) whilst NP independently assessed the qualitative and mixed methods studies, and AR independently assessed the quantitative studies. Any inter-rater disagreement was resolved through discussion.

### 2.7 Data extraction and narrative synthesis

Each study was given a unique code number for identification as follows: the qualitative studies (1QL, 2QL, 3QL…), the quantitative studies (1QN, 2QN, 3QN … ), and the mixed methods study (1MX, 2MX, 3MX … ). Relevant data were extracted from the selected studies which included: general information (i.e., authors, date, country of origin), study design, data collection method, number of participants, sex, and age.

The synthesis was carried out by HM and reviewed step by step by PD and NP. For the qualitative studies and the qualitative part of the mixed methods studies, the thematic meta-synthesis proposed by Thomas and Harden (2008) was employed. The process started by reading and rereading the included studies’ results section in detail. Then all participant quotes provided within the original studies were analysed and coded. For each article, the codes were organised and categorised to form initial themes (i.e., descriptive themes). Then the summaries of the descriptive themes of all articles were analysed iteratively and categorised to develop higher-level themes (i.e., the analytical themes). The TDF was acting as a framework to develop the coding of themes.

For the quantitative studies and the quantitative part of the mixed methods studies, meta-analysis was not undertaken as the included studies were not sufficiently homogeneous in terms of the participants involved and the outcomes (Haidich, 2010). Instead, findings relevant to the current study aims have been extracted and summarised.

To integrate the findings from the analysis of all the studies (qualitative and quantitative findings), all the findings have been combined in an overarching conceptual model which has been developed using the frameworks of the COM-B model and the TDF. This model represents the reasons that could drive people to buy prescription medicines online.

## 3 Results

### 3.1 Search results

**FIGURE 3:**
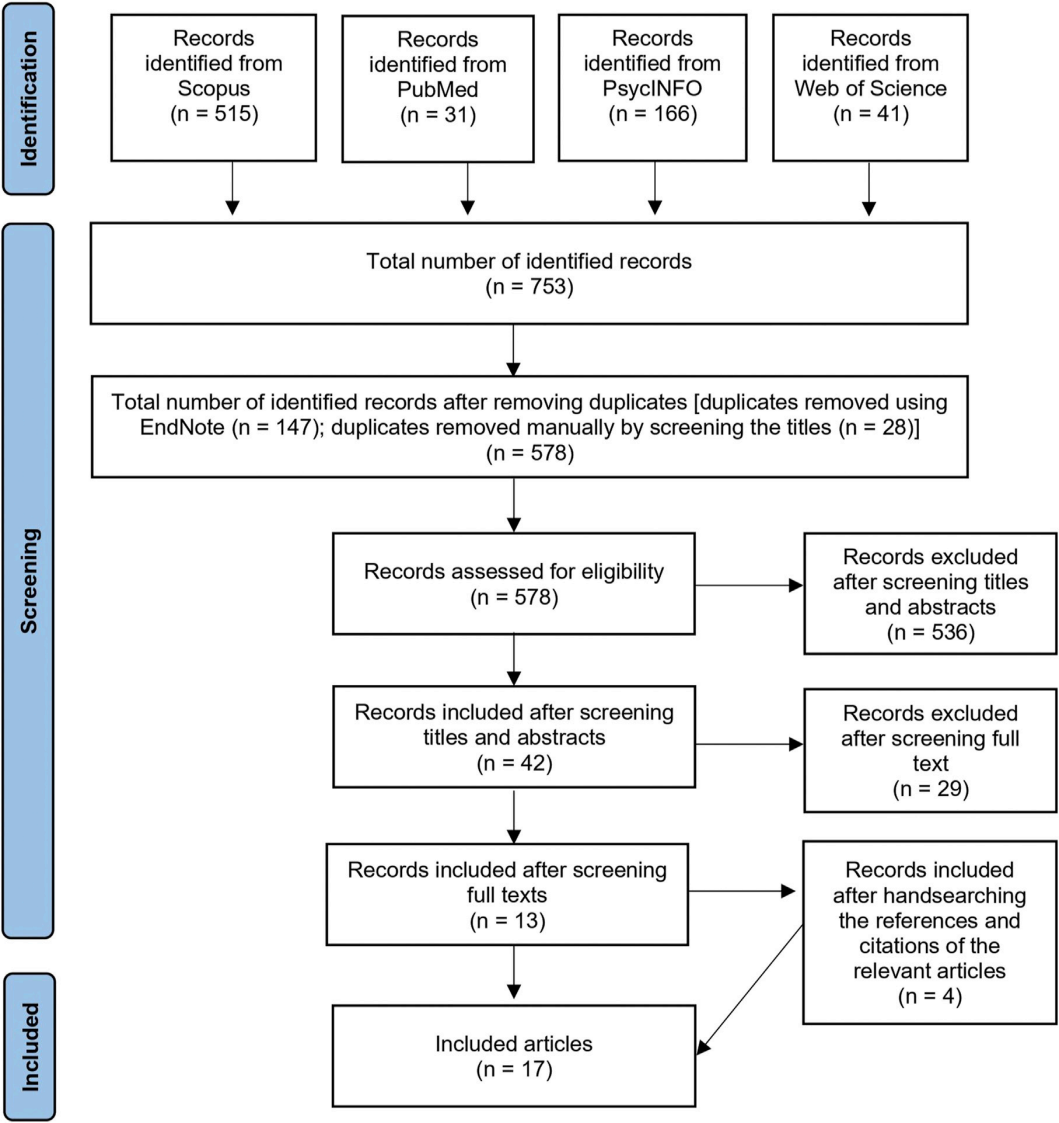
Flow diagram of the selection process.

The initial search yielded 753 articles, of which 175 were identified as duplicates. The titles and abstracts of the remaining 578 papers were reviewed, of which 536 were excluded because they did not meet the inclusion criteria. The full texts of the remaining 42 articles were reviewed, from which 29 articles were excluded. Hand-searching the citations and references of the remaining 13 articles yielded 4 more articles that met the current study inclusion criteria. In total, a sum of 17 articles was included in this study for the narrative synthesis (Figure 3).

### 3.2 Methodological quality assessment

The quality assessment showed that the qualitative studies and the mixed method study were all high quality. Of the 12 quantitative studies, 4 were scored as high quality, while 8 scored as medium quality. One-third of the quantitative studies only used descriptive statistics (4QN, 7QN, 8QN, 10QN) by summarising and describing the data obtained from a sample of respondents. However, none of these 4 studies have used inferential statistics to determine statistical significance and/or precision estimates. Appendices 2, 3, and 4 illustrate the detailed quality appraisal process. None of the articles were excluded based on the quality assessment stage.

### 3.3 Study characteristics

Table 1 illustrates the characteristics of the included studies. These studies have adopted various methodologies: qualitative method (*n* = 4), quantitative method (*n* = 12), and mixed methods (*n* = 1). The studies were based in Europe (*n* = 8), North America (*n* = 3), Middle East (*n* = 4), and two studies were conducted in several countries (multi-national).

**TABLE 1:** Studies characteristics.

Qualitative articles					
*Code*	*Citation*	*Location (population)*	*Design and data collection*	*Participants’ number and characteristics*	
1QL	Demant et al. (2020)	Sweden (internet users)	Online ethnography + interviews	N = 25 (5 buyers, 20 sellers)	
				Sex (M = 16, F = 4, Unknown = 5)	
				Age (18–37)	
2QL	Little et al. (2020)	Multinational (pregnant women)	Online focus groups	N = 23	
				Sex (M = 0, F = 23)	
				Age (25–45)	
3QL	Lundin and Liu (2020)	Sweden (internet users)	Qualitative survey (Pilot study)	N = 155	
				Sex (Not provided)	
				Age (Not provided)	
4QL	Aiken et al. (2018)	United States (pregnant women)	Interviews	N = 32	
				Sex (M = 2, F = 30)	
				Age (18–44)	
**Quantitative articles**					
1QN	Alwhaibi et al. (2021)	Saudi Arabia (internet users)	Web-based survey	N = 643	
				Sex (M = 51, F = 596)	
				Age (≥18)	
2QN	Jairoun et al. (2021)	United Arab Emirates (internet users)	Web-based survey	N = 420	
				Sex (M = 225, F = 195)	
				Age (≥18)	
3QN	Moureaud et al. (2021)	United States (internet users)	Web-based survey	N = 730	
				Sex (M = 465, F = 265)	
				Age (≥18)	
4QN	Ashames et al. (2019)	United Arab Emirates (internet users)	Survey	N = 528	
				Sex (M = 397, F = 131)	
				Age (18–25)	
5QN	Fittler et al. (2018a)	Hungarian (citizens using outpatient health services)	Survey	N = 1,055	
				Sex (M = 516, F = 539)	
				Age (≥16)	
6QN	Koenraadt and Van de Ven (2018)	Netherlands (internet users)	2 surveys: prevalence survey [*S-1*] and In-depth survey [*S-2*]	*S-1*	
				N = 50,848	
				Sex (M = 23,899, F = 26,949)	
				Age (≥18)	
				*S-2*: 447	
				N = 50,848	
				Sex (Not provided)	
				Age (Not provided)	
7QN	Abanmy (2017)	Saudi Arabia (internet users)	Cross-sectional study (Web-based survey)	N = 633	
				Sex (M = 196, F = 437)	
				Age (≥18)	
8QN	Assi et al. (2016)	Multinational (community pharmacy patients)	Cross-sectional study (Web-based survey)	N = 320	
				Sex (M = 227, F = 91, unknown = 2)	
				Age (≥18)	
9QN	Pál et al. (2015)	Romania (patients and customers of community pharmacies)	Survey	N = 253	
				Sex (M = 75, F = 178)	
				Age (≥14)	
10QN	Fittler et al. (2013b)	Hungarian (hospital patients)	Survey	N = 422	
				Sex (M = 170, F = 252)	
				Age (≥18)	
11QN	Cicero and Ellis (2012)	United States (tramadol users)	Web-based survey	N = 445	
				Sex (M = 156, F = 289)	
				Age (≥18)	
12QN	Svorc (2012)	Czech Republic (internet users	Web-based survey	N = 82	
				Sex (M = 43, F = 39)	
				Age (≥20)	
**Mixed Methods articles**					
1MX	Bowman et al. (2020)	Malta (internet users)	Survey + interviews	N = 444	
				Sex (M = 172, F = 264)	
				Age (≥18)	

### 3.4 Synthesis of qualitative findings

**FIGURE 4:**
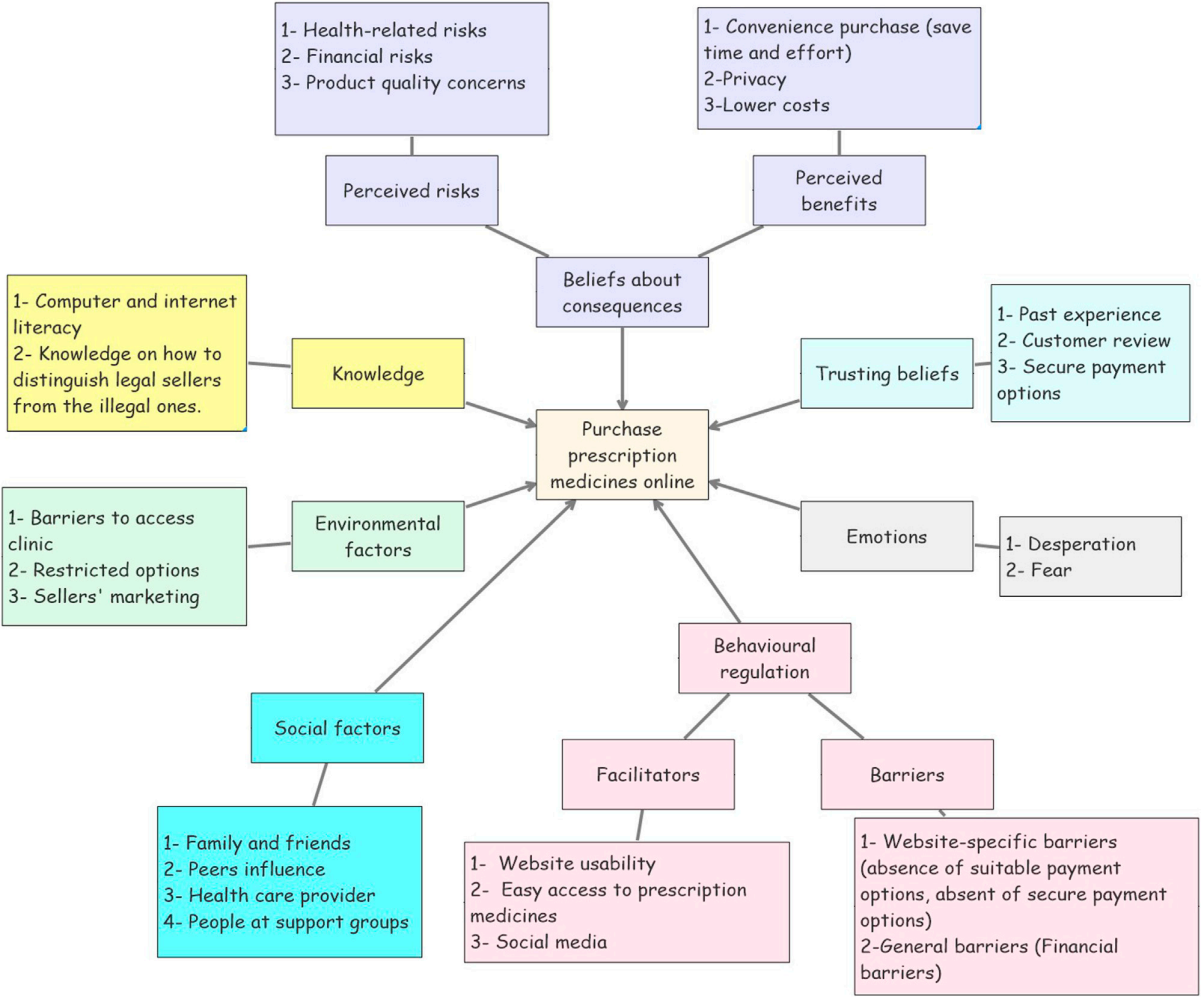
Thematic map of the qualitative findings.

The thematic analysis of the qualitative studies and the qualitative part of the mixed methods study revealed seven high-construct analytical themes (Figure 4). These themes cover consumers beliefs about the consequences of the purchase (perceived benefits and perceived risks), consumer’s emotions, factors that could increase or decrease consumer’s behavioural regulation and level of behavioural control (facilitators and barriers), consumer’s knowledge about the purchase, the antecedents that lead the consumer to trust the online sellers of medicines (trusting beliefs), social influencing factors, and the environmental factors. The TDF were used as conceptual lenses that guided the formation of the analytical themes. By applying this framework, we systematically identified and linked the factors to specific analytical themes, enabling a deeper understanding of the complex interplay between individual, social, and environmental factors driving consumers to purchase medicines on the internet.

#### 3.4.1 Benefits enticing consumer to purchase medicines on the internet

One of the analytical themes was the perceived benefits that consumers might expect if they select to purchase prescription medicines online. For example, a US-based, 38-year-old woman with three children discussed how the purchase was convenient and comfortable:

*“I wanted to stay at home with my children, and I read online there were pills now available that you can take at home if you’re quite early on. It would have been a much easier process, doing it at home, in the comfort of my own home. If they could have mailed me those pills, I could have done the abortion safely at home.”* (4QL)

Some participants discussed how people could save time when they select sourcing medicines from the internet by avoiding long waiting times to obtain appointments to see a doctor, as exemplified in the following quote:

*“There is no need to make an appointment with your GP. Well, it takes a couple of weeks to get an appointment in our surgery” (*2QL)

For one participant who has purchased abortion medicines, the privacy of the online purchase was mentioned as a factor influencing their decision:

*“I just wanted something private, convenient, and personal. For sure, it would definitely be the way to go if it was an option.”* (4QL)

Finally, low prices of medicines available online were seen as a big advantage as illustrated in the following quote:

*“I think it’s cheaper and convenience that and u can just easily go online if u know what u want and have it delivered to your door the next day”* (2QL)

#### 3.4.2 Perceived risks of purchase prescription medicines on the internet

Regarding the risks of purchasing medicines online, participants pointed out several safety concerns. Some participants mentioned the possibility of purchasing and consuming fake medicines. For example,:

*Most participants expressed concerns that online pharmacy sites were scams that would take their money and send either fake pills or an unsafe product that might cause harm […] “What if they’re not really the drugs you need? What if it’s a scam”* (4QL)

Another health-related risk perceived by the participants was the risks associated with the absence of healthcare professional oversight. One of the participants discussed how pregnant women could struggle with selecting a suitable and safe medicine if they decided to buy medicines online without involving the healthcare professional:

*“If women bypass the doctor and pharmacy it may lead to them taking medications that are unsafe in pregnancy”* (2QL)

Another risk perceived by some participants was the risk of financial scams and fraud when purchasing medicines using the Internet, as illustrated in the following quote:

*“I had to really look for the good ones. They all want to make a profit, too. What if they’re not really the drugs you need? What if it’s a scam, and they just take your money?”* (4QL)

Additionally, there was a view that medicines available online could be of inferior quality. This quote illustrates this risk:

*“…I would need to make sure it was from a source I was happy with; I would worry the quality might not be as good or it might not be what it actually says it is.”* (2QL)

#### 3.4.3 Emotions influence

Emotions also could play a role in influencing a consumer’s decision to buy prescription medicines online. People who are desperately struggling with the treatment of their illness could attempt to obtain prescription medicines online, as exemplified in the following quote:

*“… it’s such an easy market to do that in because I’m just so desperate.”* (4QL)

The fear could also play a role. Participants in one of the included studies have expressed their fear of purchasing medicines from the internet without involving their doctors, as illustrated in the following quote:

*“I would be very cautious about buying meds online, especially when pregnant. When pregnant, I am generally a little more cautious anyway. I think I would rather present my bump to a pharmacist just to reinforce that I am pregnant and to make sure the meds are suitable”* (2QL)

#### 3.4.4 Facilitators of the purchase

The next analytical theme revealed was the facilitators of the purchasing of prescription medicines from the Internet. For example, the ease of use of the website (Websites usability) where consumers could purchase prescription medicines with just a few steps and would remember passwords and payment card details for the next purchases:

*“…I can buy in three clicks. If there is difficult signing in, remembering passwords, looking for payment cards etc. I can be put off’* (2QL)

Another facilitator that was offered by the online sellers was online access to prescription medicines. The following example illustrates this point from the perspective of a pregnant woman based in the United States who has chosen to purchase abortion pills (prescription medicine that requires a prescription and medical supervision) from the internet because of the accessibility to these medicines without a prescription:

*“You can buy anything online, so my second thought was basically to order an abortion kit online”* (4QL)

Social media platforms were discussed by participants in some of the studies as a factor that could facilitate the online purchase of prescription medicines. For example, Instagram was found to facilitate communication and connection between buyers and sellers as shown in this example:

*“Instagram was perfect to establish contacts. One of the people that I came into contact with at that time is now a very good friend of mine and I make the majority of my purchases with him”* (1QL)

Furthermore, social media platforms hosted different support groups which could provide advice on what medication was helpful:

*“Mostly, the posts are “does anybody sell […] amphetamine, etc.” ]In some of these groups, there have also been posts like, “Where in [a location] in the city can one buy this and that drug?” and like, asking about where it’s safest to sell, and people ask for advice and stuff like that.”* (1QL)

#### 3.4.5 Barriers to purchasing prescription medicines on the internet

The barriers to purchasing medicines online have been classified into barriers that either prevent people from buying medicines from a specific website (Website-specific barrier) or barriers from the internet in general regardless of which website they could select (General barrier) (Almomani et al., 2023a). Website-specific barriers include the payment method suitability and security. Absent of a suitable payment option could delay the purchase, as exemplified here:

*“There was a handful of websites, and a couple I tried to order from, but they had overseas banks, and my bank would not work to give them money. So, I just started thinking they were sketchy as hell.”* (4QL)

Another website-specific barrier was website security. For example, the absence of a secure payment method was highlighted as a factor that could hinder the online purchase:

*“…if PayPal is an option, I tend to trust the website. I know there is a backup if something goes wrong and also its PayPal which has my information and not the actual website.”* (2QL)

General barriers included the financial capability of the consumer, as shown in the following example:

*“The costs (around $250–300 for a mifepristone-misoprostol combination pack) out of reach.”* (4QL)

#### 3.4.6 Knowledge about the purchase

This superordinate theme represents consumers’ knowledge about the procedure of purchasing prescription medicines online as a factor that could influence a consumer’s decision to make the purchase. One of the studies found that more internet-confident consumers who had the knowledge and experience (computer and internet literacy) were more likely to purchase prescription medication online:

*“…the younger population would be more accepting … mostly because they are more likely to have done online purchasing before but with other products.”* (2QL)

Some participants discussed the difficulties in recognising legal online sellers from illegal ones. The following example illustrates this point from the perspective of one participant who express scepticism against the logo used to distinguish legal sellers from illegal ones:

*“It feels too easy to plagiarize and misuse logos on the Internet.”* (3QL)

#### 3.4.7 Trusting beliefs

This superordinate theme includes the factors that influence consumers’ trust in online sellers of medicines. For example, some consumers trust websites they are already familiar with. Therefore, consumer’s past experience could play a role in the decision to purchase prescription medicines online, as shown in the following example:

“…I would probably only use a company that I am already familiar with.” (2QL)

Other customer’s feedback and evaluation (whether negative or positive) of medicines they have purchased and used or had experience with (i.e., customer review) were highlighted as a factor that could lead people to trust the online seller of medicines:

“would not look twice unless there were quite a number of reviews and obviously the majority positive. I would be swayed by any negative reviews to avoid purchasing.” (2QL)

The availability of secure payment options such as PayPal at online seller’s websites could also influence trust, as this kind of payment system offers protection for consumers against fraud by offering a refund in case a scam happened. The following example illustrates this point:

“…if PayPal is an option, I tend to trust the website. I know there is a backup if something goes wrong” (2QL)

#### 3.4.8 Social influencing factors

Social factors were found to play a role in influencing a consumer’s decision to purchase medicines online. These factors were family and friends, peers with similar experiences, and healthcare providers (doctors or pharmacists). The following quotes illustrate these factors:

*“…Family and friends probably would advise against if the purchase would be made without doctor’s approval or consent”* (2QL)

*“Some of the women in the group felt they would be judged by their peers for purchasing medication online during pregnancy […] ‘…I do not think I would ask others as part of me thinks it sounds stupid so therefore it’s wrong others would then think I was being foolish and judge me. But as others have said, if it was more common practice among people I know, then I would not be so wary of it.”* (2QL)

*“This in turn gives the basis for building a relationship with the care providers and subsequently trusting them with knowledge about medicines. This trust is important in determining the source of purchase of medicines making online purchase from unknown sources less likely. Participants expressed the trust they have in their doctor or pharmacist “No I ask a lot but do not forget I (.) have a health background and I have an enormous respect towards the pharmacists I understand that their course is as long as that of a doctor and is more focused on medicines and so I take their opinion…”* (1MX)

In one of the studies, people at support groups available on social media were also pointed out as a factor that plays a role in influencing consumer’s decision to buy prescription medicines online:

*“Most pregnant and new moms are in social media groups where they have access to multiple opinions and suggestions regarding symptoms of pregnancy and new-borns. in a situation of discomfort they may be induced to buy online without doctor / pharmacist opinion.”* (2QL)

#### 3.4.9 Environmental factors

This theme includes any environmental condition that discourages or encourages the behaviour of purchasing prescription medicines via the Internet.

One of the environmental conditions that could trigger people to buy medicines online is the difficulty to access the clinics which could be financial or logistical difficulties. The following quote is the perspective of a 35-year-old woman who turned to obtain abortion pills online because she could not afford the abortion clinic fees.

*“After I’d made my decision to end the pregnancy, I did not know where the money could come from. It was going to have to come out of my rent money. So, for me, it was a matter of having to decide: Pay my rent or pay for the abortion…. So, I just typed in ‘abortion pills’ online, and then I looked on social media”* (4QL)

Another condition that could encourage people to buy prescription medicines online is the restricted options available in local market sectors where few alternatives are available. For example, the public healthcare system in Malta procures medicines on the ‘cheapest compliant with specifications’ principle (Bowman et al., 2020), and consequently, only a few options could be available for the patient:

*“I needed Oxis but they do not bring Oxis. They bring a lesser quality”* (1MX)

One more encouraging condition was the marketing used by the online sellers of medicines. Online sellers of medicines were trying to persuade consumers to make the purchase by providing an environment and services that make the purchase easier for consumers such as advertising or influencer marketing as exemplified here:

*“Recommendations and advertising play a big role. Pregnancy is a time when I feel women will do anything to ensure the health of her baby, so if adverts or other mothers say a product is the best then the woman will want to purchase it”* (2QL)

### 3.5 Synthesis of quantitative findings

The findings of the quantitative studies that are relevant to the current study aim were extracted and summarised in Appendix 5. Twelve cross-sectional survey studies in addition to the quantitative part of the mixed-method study were explored. These studies included data from a total of 6,422 participants living in different countries. These studies included views from people who have purchased medicines online in the past (including prescription medicines) and/or people who did not purchase medicines online previously but have views and opinions about this kind of purchase. Several types of prescription medicines were purchased (e.g., antibiotics, controlled medicines, and COVID-19 vaccines).

In Appendix 5, we summarised the types of medicines purchased and the factors that could influence a consumer’s decision of purchasing prescription medicines via the Internet.

### 3.6 Integration of the findings

**FIGURE 5:**
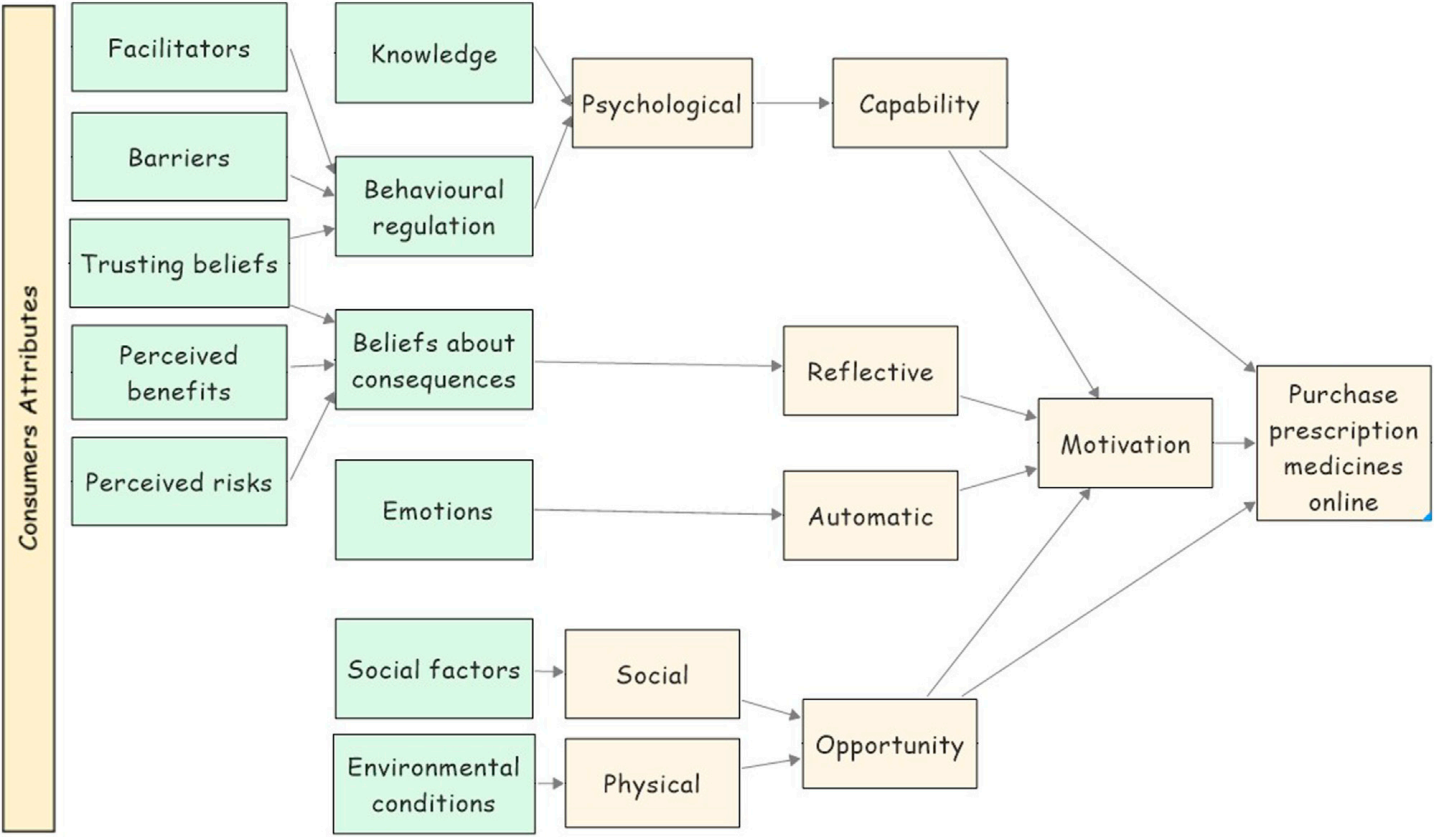
Factors that could influence consumer’s decision of buying prescription medicines online mapped against the COM-B model and the TDF.

To integrate the findings from the qualitative and quantitative studies, the findings have been categorised against the COM-B model. Relying on this theory, our study proposes an overarching conceptual framework that represents the factors that could influence consumer’s decision to purchase prescription medicines using the Internet (Figure 5).

Using Figure 2, six of the analytical themes (knowledge, emotions, behavioural regulations, beliefs about consequences, social factors, and environmental factors) were mapped directly against the COM-B model. The last analytical theme (i.e., trusting beliefs) holds a unique position as it inherently intersects with both domains, the belief about consequences and the behavioural regulation domains. Trust has been placed as ‘a belief about consequences’ as it enables consumers’ positive expectation that no negative consequences will happen to them, thus creating a favourable perception of the outcomes (Pavlou and Fygenson, 2006; Almomani et al., 2023a). Concurrently, trust has been also proposed as a behavioural regulation factor, as it builds consumers’ confidence to depend on the online sellers of medicines, which helps consumers overcome psychological barriers to engaging in the behaviour (Pavlou, 2002; Almomani et al., 2023a). Thus, increasing consumers control over the behaviour. This dual placement offering a comprehensive understanding of how trust influences cognitive appraisal and self-regulation, thus shaping the behaviour."

Figure 5 highlights the superordinate themes revealed from the analysis, however, within these themes, a breadth of specific details has also been produced. Table 2 illustrates these superordinate themes and their compositions, as well as the source in which each of these themes was cited.

**TABLE 2:** The overarching table which includes the themes identified from the synthesis and their compositions.

Reasons why people purchase prescription medicines on the internet. ^(sources)^
• Perceived benefits: Positive consequences and outcomes that consumers expect to receive from purchasing prescription medicines on the Internet.
⁃ Convenience purchase: Save effort, avoid long waiting times, helpful when the patient is in a bed rest period, fast deliveries, and 24/7 accessibility. ^(2QL, 3QL, 4QL, 1QN, 5QN, 6QN, 7QN, 8QN, 1MX)^
⁃ Privacy. ^(2QL, 4QL, 1QN, 7QN, 11QN)^
⁃ Lower cost. ^(2QL, 3QL, 4QL, 1QN, 3QN, 4QN, 5QN, 6QN, 8QN, 9QN, 10QN, 11QN, 12MX)^
⁃ Bypassing gatekeepers: Bypass doctors who refuse to prescribe prescription medicines. ^(11QN)^
⁃ Medicines availability
- Availability of extra quantities. ^(3QN)^
- Availability of medicines that are not available locally. ^(1QN, 4QN, 5QN, 6QN, 8QN, 1MX)^
- Being able to purchase unlicensed medicines. ^(3QN, 8QN)^
- Wide products choices. ^(1QN, 4QN, 8QN)^
- Brand medicines availability. ^(3QN, 1MX)^
⁃ Better product quality. ^(1QN, 4QN, 5QN, 6QN, 8QN, 9QN)^
⁃ Avoid withdrawal symptoms for patients using controlled medicines. ^(11QN)^
• **Perceived risks:** Negative outcomes associated with purchasing prescription medicines using the Internet.
⁃ Health risks
- Possibility of purchasing fake medicines. ^(4QL, 5QN, 1MX)^
- Absence of medical oversight complications, for example, the possibility of misusing medicines. ^(2QL, 3QN, 5QN, 9QN)^
⁃ Financial risks. ^(4QL)^
⁃ Inferior product quality. ^(2QL, 4QN, 5QN, 7QN, 9QN, 1MX)^
⁃ Engaging in illegal behaviour (buying from Unlicensed websites or buying unlicensed medicines). ^(4QN, 7QN)^
⁃ No privacy and confidentiality. ^(7QN)^
⁃ Delayed deliveries. ^(5QN)^
• **Emotions:** A complex reactions, feelings, and affective states that motivate consumers to purchase prescription medicines from the internet
⁃ Desperation caused by medication unavailability. ^(4QL)^
⁃ Fear of the purchase risks. ^(2QL)^
⁃ Hate going to the doctor. ^(11QN)^
• **Facilitators:** Factors that increase consumers’ ability to control the behaviour of purchasing prescription medicines using the web, thus, facilitating the purchase
⁃ Facilitators offered by the online sellers of medicines that make it easier for customers to purchase their product
- Websites usability. ^(2QL)^
- Easy accessibility as prescription medicines without requiring a prescription. ^(4QL, 1QN, 3QN, 4QN, 6QN, 8QN, 9QN)^
- Fast shipping option. ^(7QN)^
- Refill reminders by email. ^(7QN)^
- Products can be compared faster. ^(5QN)^
- Product information and providing instruction on how to use prescription medicines. ^(1QN, 5N, 7QN, 8QN, 10QN)^
- Online sellers marketing such as promotion and discounts which make the purchase affordable to consumers. ^(1QL, 2QL, 10QN, 12QN, 1MX)^
⁃ Facilitators offered by social media
- Easy and private communication. ^(1QL, 3QN)^
- Act as a source of medical information offered by the support groups available on the different social media platforms. ^(1QL, 2QL)^
• **Barriers:** Factors that decrease consumers’ ability to control the behaviour of purchasing prescription medicines using the web, thus, impeding the purchase
⁃ Website-specific barriers (barriers that delay the purchase until consumers can find an alternative)
- Absent of suitable payment option. ^(4QL)^
- Payment method security. ^(2QL)^
- Website language and instructions are in an unknown foreign language. ^(3QN)^
⁃ General barriers (barriers that prevent the purchase)
- Financial capabilities. ^(4QL)^
- Consumers do not know how to use the medicine. ^(3QN, 5QN)^
• **Knowledge:** Consumer’s knowledge about the purchase (procedural knowledge and the knowledge about the risks of the purchase)
⁃ Computer and internet literacy. ^(2QL)^
⁃ Knowledge about the risks of the purchase. ^(2QL, 3QL, 3QN, 10QN, 12QN)^
⁃ The difficulty in distinguishing between legal and illegal online pharmacies. ^(3QL, 3QN)^
• **Trusting beliefs:** Factors that lead consumers to trust the online sellers of medicines
⁃ Past purchase experience (either positive or negative experience). ^(3QN, 6QN, 7QN, 12QN, 1MX)^
⁃ Customer review (either positive or negative review and feedback). ^(2QL, 4QL, 6QN, 12QN, 1MX)^
⁃ Availability of secure payment option. ^(2QL)^
⁃ Clear information about the product and vendors. ^(6QN, 7QN)^
• **Social influencing factors:** Social factors that could encourage or discourage consumers’ decision to purchase prescription medicines using the Internet.
⁃ Family and friends. ^(2QL, 3QN, 6QN, 12QN, 1MX)^
⁃ Peer influence: People who are in the same situation. ^(2QL)^
⁃ Healthcare provider: Doctors or pharmacists. ^(2QL, 3QN, 1MX)^
⁃ People at online support groups. ^(2QL)^
• **Environmental factors:** Any external environmental condition that encourages or discourages the purchase
⁃ Barriers to accessing the clinic (logistical difficulties, financial difficulties). ^(4QL)^
⁃ Restricted options (few alternatives are available in local market sectors). ^(1MX)^
⁃ Sellers’ marketing (advertising and pop-up ads). ^(2QL)^
⁃ Medicines shortages. ^(8QN)^
⁃ Unsatisfied with the quality of clinical services provided in clinics or local community pharmacies. ^(1QN, 1MX)^
⁃ The Coronavirus pandemic (COVID-19). ^(2QN)^
• **Consumer attributes:** Demographic, socioeconomics, health information, and the internet using habits and characteristics that could affect the purchasing decision
⁃ Age. ^(1QN, 2 QN, 4 QN, 5QN, 6QN, 7QN, 11QN, 1 MX)^
⁃ Sex. ^(1QN, 2QN, QN, 7QN)^
⁃ Marital status. ^(2QN)^
⁃ Income level. ^(1QN, 7QN)^
⁃ Educational level. ^(1QN, 2QN, 3QN, 4QN, 5QN, 7QN, 1MX)^
⁃ Employment. ^(1QN, 3QN)^
⁃ Health insurance availability. ^(11QN)^
⁃ Time spent on the internet. ^(5QN, 10QN)^
⁃ Social media affinity. ^(3QN)^
⁃ Internet purchase frequency in general. ^(5QN)^

## 4 Discussion

### 4.1 Principal findings

#### 4.1.1 Prevalence of people purchasing medicines online

Despite the abundance of public awareness campaigns that warn consumers about purchasing prescription medicines on the Internet (ASOP, 2020), the evidence explored in the current study found that people are buying medicines from the Internet and put themselves at risks associated with this purchase.

According to the quantitative studies’ findings which are shown in Table 3, the percentage of people purchasing medicines using the Internet varies as explored data differs, due to the type of product purchased and study location. Of the 12 quantitative studies explored, 11 studies provided the number of consumers who purchased medicines using the Internet. Of the 5,896 participants explored in these 11 studies, 1,123 (19%) have purchased medicines (either over-the-counter medicines or prescription medicines) from the Internet.

**TABLE 3:** Prevalence of people purchasing medicines using the Internet (N*: number of participants).

Citation	N*	Prevalence of people who purchase medicines online	Medicines purchased
Alwhaibi *et al.* (2021)	643	235 (36.5%) participants bought medicines over the Internet.	Viagra, birth control bills, antibiotics, narcotics, refill medication for chronic conditions, herbal medicine, supplements, and cosmetics
Jairoun et al. (2021)	420	131 (31.2%) participants bought medicines over the Internet.	Analgesics, Antihistamines, Anti-cough medicine, dietary supplements
Moureaud *et al.* (2021)	730	131 (17.9%) participants bought medicines over the Internet.	Sedatives (Xanax^®^, Valium^®^, Ativan^®^, *etc.*), stimulants (Adderall^®^, Ritalin^®^, *etc.*), narcotics (Vicodin^®^, Percocet^®^, Oxycontin^®^, fentanyl, *etc.*), and COVID-19 medicines or vaccines
Ashames *et al.* (2019)	528	53 (10%) participants bought medicines over the Internet.	Prescription medicines + over-the-counter medicines
Fittler *et al.* (2018a)	1,055	44 (4.17%) participants bought medicines over the Internet.	Prescription medicines + over-the-counter medicines
Koenraadt and Van de Ven (2018)	447	153 (34.3%) participants bought medicines over the Internet.	Prescription medicines
Abanmy (2017)	633	17 (2.7%) participants bought medicines over the Internet.	Prescription medicines + over-the-counter medicines
Assi *et al.* (2016)	320	208 (65%) participants bought medicines over the Internet.	Prescription medicines + over-the-counter medicines
Pál et al. (2015)	253	21 (8.3%) bought medicines over the Internet.	Prescription medicines + over-the-counter medicines
Fittler *et al.* (2013b)	422	34 (8.1%) bought medicines or dietary supplements over the Internet.	Prescription medicines + over-the-counter medicines
Cicero and Ellis (2012)	445	96 (21.6%) bought tramadol online without prescription	Tramadol
Svorc (2012)	82	Not provided	Prescription medicines + over-the-counter medicines

#### 4.1.2 Antecedents of purchasing prescription medicines on the internet

This systematic review provides a comprehensive overview of the factors that could influence people’s decisions to purchase prescription medicines from the Internet. These factors include the perceived benefits that entice consumers to make the purchase, perceived risks associated with the purchase, consumer’s emotions, facilitators that can make it easier for the consumer to regulate and control their behaviour, barriers that can decrease consumer’s level of control over their behaviour, trusting beliefs that help make the consumers trust the online sellers of medicines, social influencing factors, external environmental factors that could trigger consumers to involve in the purchase, and consumers knowledge of the purchase.

##### 4.1.2.1 Evaluating the benefits and risks of the purchase

The current review study summarised what consumers perceived about the benefits and the risks associated with purchasing prescription medicines from the Internet. Consumers might be enticed by several benefits of the purchase (e.g., lower prices, privacy, saving time and effort, and bypassing gatekeepers). However, many risks were also associated with the purchase (e.g., health-related risks and financial risks) which could affect the purchasing decision and discourage consumers from making the purchase.

Before making the purchase, consumers try to evaluate what they perceive about the consequences of the purchase. This evaluation process is a complex cognitive process in which consumers take into consideration what they perceive about the positive and negative outcomes of the purchase, so if the positive outcomes (benefits) outweigh the negative ones (risks), then there might be a preference to make the purchase. Additionally, the probability of making the purchase could be increased when consumers have inadequate knowledge about the risks associated with the purchase.

##### 4.1.2.2 Emotions influence

The cognitive process discussed above (i.e., evaluating the risks and benefits of the purchase) focuses on the rational part of the decision-making by assuming that consumers are rational in thinking. However, consumers’ emotional status could play a role and bias consumers’ judgments and choices (Ajzen, 2020), which could make them behave irrationally even in the case they perceive that the risks of the purchase outweigh the benefits. The evidence explored in this review found that desperation could drive consumers to behave without conscious thought, meaning that when desperate buyers, who are struggling with obtaining prescription medicines for a serious medical condition, find the medicines available online, then the purchase could be more likely to occur even if the buyers were aware of the risks of the purchase.

##### 4.1.2.3 Healthcare provider role

The current review found that healthcare providers can play a harmful or beneficial role in influencing consumer’s decision to purchase prescription medicines from the Internet. So, when the healthcare provider refuses to prescribe a specific medications to the patient who think that this medication is useful to them, then patients might seek for alternatives including the internet (Cicero and Ellis, 2012), thus, putting the patient at risk of end up buying fake medicines which are widely available on the internet (Almomani et al., 2023a). On the other hand, healthcare providers could play a beneficial role by educating patients to about the risks of purchasing prescription medicines from the internet and how to purchase medicines safely (Bowman et al., 2020). This finding supports a finding from a survey conducted in the United States by ASOP which found that healthcare providers could influence a consumer’s decision about purchasing medicines online by educating consumers about how to buy medicines online safely (ASOP, 2020).

##### 4.1.2.4 The facilitating role of social media platforms and encrypted messaging apps

The current study also summarised the facilitators and barriers to purchasing prescription medicines online. Consumers could face barriers that might delay or prevent purchasing medicines from the internet such as the complex instruction for using some prescription medicines, however, social media platforms could facilitate the purchase and help consumers overcome this barrier as these platforms host different support groups which could provide assist consumers by providing them with the information about how to use these prescription medicines. As a result, consumers’ self-efficacy and control over their behaviour will increase, which in turn could drive consumers to purchase prescription medicines without involving the healthcare providers and without any medical supervision.

Additionally, the social media platforms (such as Facebook, Twitter, and Instagram) and encrypted messaging applications (such as WhatsApp or Telegram) can play a role as a private communication channel between the buyers and sellers of prescription medicines. The messaging applications could offer the option of end-to-end encryption to protect user privacy. End-to-end encryption is a type of encryption that is adopted by some messaging applications to ensures that only both parties (the sender and the receiver of a message) can read the message contents (Kulshrestha and Mayer, 2021). Thus, these applications offer a private and secure means of communication between buyers and sellers, which could help alleviate concerns about the potential risks of engaging in this illegal behaviour.

##### 4.1.2.5 Easy access to prescription medicines without requiring a prescription

Another facilitator offered by the online sellers of medicines highlighted in this review was the accessibility to prescription medicines without the need for a prescription. Several previous studies have found that many prescription medicines available on the internet and easily accessible for anyone without requiring a prescription include high-risk controlled medicines and antibiotics (Boyd et al., 2017; Monteith and Glenn, 2018; Hockenhull et al., 2020). The current study findings are in line with these studies, as this practice (offering prescription medicines requiring a prescription) have facilitated the purchase and made it easier for consumer to control the purchase, thus, consumers can purchase different types of prescription medicines (including controlled medicines and antibiotics) easily and without limits. This is problematic as the easy access to controlled medicines could increase the possibility of abusing the medicines. Moreover, buying antibiotics online without medical oversight could increase antimicrobial resistance (Boyd et al., 2017).

##### 4.1.2.6 Medicine shortages and purchasing prescription medicines on the internet

One important environmental condition highlighted in this review is the impact of the medicine shortages on purchasing prescription medicines from the Internet. Medicine shortages can be caused by many reasons including the sudden increase in demand for a specific medication, supply side problems (manufacturing obstacles such as the shortages of the raw material), pandemics such as the Coronavirus disease (COVID-19), political events (such as the Brexit), or government policies such as the price controls which make it unprofitable for pharmaceutical companies to manufacture certain products (Iyengar et al., 2016; Musazzi et al., 2020; Badreldin and Atallah, 2021). Medicines shortages can frustrate consumers as they might struggle to obtain the medicines they need, which could create a sense of desperation for consumers, which in turn could encourage them to seek alternative sources including the Internet to obtain their needs. This is problematic as previous studies have shown that medicines in shortage are widely available online without requiring a prescription or healthcare provider involvement. For example, a study conducted in Europe found that anticancer drugs affected by shortages were available and accessible online without medical prescription. While another study conducted in the United States found that vaccines in shortage are widely available and easily accessible on the Internet (Liang and Mackey, 2012; Fittler et al., 2018b). Thus, this availability and easy accessibility of prescription medicines on the internet could encourage desperate consumers to make the purchase. Moreover, this finding is in line with a recent study that analysed the news media coverage of the problem of purchasing prescription medicines on the internet (Almomani et al., 2023b), in which the medicine shortages were highlighted as a condition that encourages people to people to purchase prescription medicines online.

##### 4.1.2.7 Customer review impact on the purchase

Another factor found that can influence a consumer’s decision to purchase prescription medicines online is consumer trust in online sellers. Trust was found to be an important determinant of consumers’ purchasing behaviour in the e-commerce context (Pavlou, 2002; Bourlakis et al., 2008). Some of the participants in the studies included in this review trusted websites they had previously purchased from without facing any problems, while others judged whether a website was trustable or not by relying on other customer reviews. This is in line with the findings from a review study that found that pregnant women were influenced by customer reviews (Little et al., 2018). Customer review represents other consumers’ feedback on a specific product or purchase and is found to be one of the relevant sources of information that could affect consumer’s decision of purchasing products online (Yaylı and Bayram, 2012). Thus, these reviews could have a substantial effect on consumer’s decision of purchasing prescription medicines on the Internet This is worrying because the illegal sellers of medicines could use this tool (i.e., customer review) by creating fake positive reviews that increase the reputation and rating of their website, thus make the illegal seller’s websites more trustable and credible.

#### 4.1.3 Problem complexity

We found that consumer behaviour of purchasing prescription medicines from the internet is a complex and multi-dimensional phenomenon that is influenced by a range of internal and external factors. This complexity arises from the multiple factors that interfere with the purchasing decision including the cognitive process, affective process, social influencing factors, external environmental factors, and economic factors. Consumer’s decisions about purchasing prescription medicines from the Internet could be influenced by cognitive processes such as perception and knowledge. However, other factors including emotions could bias consumer judgments and behaviour, which in turn increases the complexity of understanding the decision. Moreover, several external factors such as the social influencing factors and the environmental factors that are out of the consumer’s control could also influence the consumer’s decision. What added further complexity to consumer’s decision is the difference between consumers in terms of their cultures and countries which could have different healthcare systems and different regulations and legislations.

The overarching interpretation of the consumers behaviour of purchasing prescription medicines from the internet provided by the current theory-based systematic review enables a deeper understanding of this complex behaviour and decision-making process, thus, this review provides the basis for policymakers and regulators to take effective actions that can protect consumers from the risks of such purchase.

### 4.2 Strength and limitations

This study is the first systematic review that looks solely at the consumers behaviour of purchasing prescription medicines via the Internet. This review provided an overarching view of the breadth of reasons that lead people to buy prescription medicines from the internet. This review provides diversified views about the research topic as the included studies were conducted in different countries and the participants were from diverse cultures, sex, and age groups. Furthermore, the studies included in this review used different methodologies (qualitative, quantitative, and mixed methods), thus, providing stronger evidence and more confidence in the review findings. In addition, this review was guided by the COM-B model and the TDF theories, and these behavioural theories were validated by research in different contexts, thus, increasing the validity and credibility of the research findings.

One limitation of this study is that the non-English language articles were excluded which might affect the generalisability of the study. Another limitation is that the heterogeneity among the quantitative studies precluded a meta-analysis, therefore, the method we employed to synthesize their findings could not generate effect size estimates, thus, it cannot assess the magnitude of the purported relationship between the independent variables (factors that influence buying behaviour) on the dependent variable (buying prescription medicines online). However, a questionnaire could be developed, in the future, based on the study findings to measure the weight of the factors that influence consumers’ decisions of purchasing medicines online to evaluate their relevance. Finally, the sample population of half of the included qualitative studies focused only on pregnant women’s point of view which could limit the generalisability of the qualitative findings.

### 4.3 Implications for regulators and policymakers

#### 4.3.1 Evidence-based consumers education is needed

Several awareness campaigns have been run to warn consumers about the risks of purchasing prescription medicines from the Internet (ASOP, 2020). However, our systematic review founds that people still obtain their medicines from the internet and put themselves at risk associated with this purchase.

To improve those awareness campaigns’ effectiveness, we recommend the development of evidence-based campaigns relying on scientific knowledge (such as the current systematic review) in order to improve the effectiveness, outcomes, and cost-effectiveness of these campaigns. For example, tailored messages focusing on the high costs incurred if failure of treatment occurs, due to the use of ineffective medicines, can be developed to target consumers seeking cost saving options, while other messages focusing on the health risks (caused by possible drug-drug interactions or the risks of fake medicines) can be developed to target individuals with chronic medical conditions. Additionally, as this review found that the healthcare providers could influence consumer’s decision of purchasing medicines online, then the active engagement with healthcare professionals (doctors and pharmacists) in the awareness campaign could be beneficial by encouraging safer medication practices.

#### 4.3.2 Long waiting times to receive the treatment

Based on our findings, one of the factors that could play a role in increasing the purchase of prescription medicines from the Internet is the long waiting times that patients have to wait in order to receive their treatment. The long waiting times can be frustrating and can negatively impact patients’ health and outcomes (Reichert and Jacobs, 2018). This is a long-standing problem and difficult to solve. As patients continue to face long waiting times for medical consultations, their tendency to resort to medicine purchases through the internet may further escalate. Thus, a holistic approach is needed to effectively resolve this problem by involving healthcare system improvements, policy interventions, technology, and innovative solutions to ensure timely access to quality healthcare for all people.

To address this problem, healthcare process improvement is needed by assessing and evaluating the processes to identify any areas that may be causing delays in order to eliminate unnecessary stages and increase the efficiency of the process. For example, regulators in healthcare sectors could check if the healthcare providers and administrators handle the high workload efficiently, if not, then provide suitable training. In the case that staff shortages are the problem, then increasing the capacity could be the solution by hiring more medical staff, and in case this is not applicable, then regulators and policymakers could increase the number of authorised prescribers (people authorised to prescribe the prescription medicines) by authorising pharmacists to prescribe prescription medicines for certain conditions or in certain situations.

#### 4.3.3 The accountability problem should be addressed

The current study found that different Internet platforms including social media, search engines, encrypted messaging applications, and customer review websites have facilitated the purchase of prescription medicines from the internet. As a result, consumer’s life might be at risk due to the misuse of medicines or consuming fake medicines that are widely available online, while the government might incur more costs to combat this kind of sale. Moreover, the pharmaceutical companies could also incur financial losses caused by the drop in sales.

The only gainers of this purchase are the illegal online sellers of medicines who could obtain a profit margin of more than 7,000% from selling the high profit fake medicines (OECD/EUIPO, 2020), and the internet platforms which host those sellers, as these platforms (social media, search engines, customer review websites) could gain from driving traffic and user engagement to their platforms, as the illegal sellers of medicines can drive traffic these platforms by offering prescription medicines that are not easily available through legitimate channels. Therefore, given the serious patient safety concerns associated with this purchase, policymakers and regulators must hold a higher level of accountability and responsibility to these platforms to prevent such activity.

### 4.4 Research gaps and future research agenda

#### 4.4.1 Context gaps

Regarding the geographical coverage, the majority of the research was conducted in Europe (47%, *n* = 8), these studies were conducted in Romania, the Czech Republic, Hungarian, Malta, the Netherlands, and Sweden. However, to the end of 2021, there are shortages in studies conducted in several countries in Europe such as Germany, Spain, France and the United Kingdom. Whereas in Asia, only 4 studies (23.5%) were conducted, and all of these studies were based in the Middle East.

Given the limited research that analyse the consumer behaviour of purchasing prescription medicines from the internet in several countries, such as Australia, Canada, United Kingdom, China, South America, Africa, and several other countries. Future studies are needed to address this gap and provide a more comprehensive understanding of this phenomenon from a global perspective.

Regarding the included studies’ populations. The population of half of the qualitative studies included in this review was pregnant women, which in turn, could limit the generalisability of the findings. Thus, further qualitative studies that explore populations other than pregnant women could help in covering this gap.

#### 4.4.2 Intervention studies are needed

This systematic review has identified the breadth of reasons that drive people to purchase prescription medicines via the internet, meaning that the current study identified what needs to be changed in order to design interventions to minimise the purchase of prescription medicines from the internet. Thus, the current study findings could provide the basis for future researchers to conduct intervention studies that aim to promote positive changes in behaviour and develop behavioural intervention techniques in order to minimise the purchase of prescription medicines from the internet, thus protecting consumers from the risks of the purchase including the risks fake medicines.

#### 4.4.3 The use of behavioural theories

The behaviour of purchasing prescription medicines from the internet is complex and not straightforward to understand. However, underpinning the research using validated behavioural theories could help in understanding complex human behaviour as theories enable researchers to look at data from different angles within which to conduct the analysis and defined which key variables influence a phenomenon of interest (Reeves et al., 2008). Growing evidence supports the use of theory when understanding behaviour as theories could provide tentative explanations for why and under what circumstances behaviours occur (Alhusein et al., 2021).

The majority of the studies explored in this review did not adopt any particular theoretical frameworks. Only 2 articles (11.77%) in the included studies explored the phenomenon using behavioural theories (i.e., the theory of planned behaviour, and the technology acceptance model) to underpin the research (Svorc, 2012; Little et al., 2020). Thus, further studies, which use behavioural theories to interpret the consumer behaviour of purchasing prescription medicines from the Internet, will need to be undertaken.

#### 4.4.4 Methodological gaps

Most of the included articles that explored consumer behaviour of purchasing prescription medicines from the Internet adopted a quantitative approach by using surveys (%70, *n* = 12) to collect data. While surveys are widely used for data collection, researchers should be cautious about the limitations of this method as it may lead to an inaccurate understanding of participants’ experiences, as it lacks the depth provided by more qualitative approaches like interviews (Taherdoost, 2022). Meantime, the limited response options in surveys may not capture the full range of possible answers of participants’ opinions, leading to oversimplification of complex issues.

As shown in Table 1, only a few studies to date employed qualitative methods including individual interviews, focus groups, ethnography, and qualitative questionnaires. Qualitative research generates rich data and enables a deeper understanding of this phenomenon (Bell et al., 2022). Future studies which use qualitative research methods on the current phenomenon in different research contexts are therefore recommended.

## 5 Conclusion

This systematic review of qualitative and quantitative studies explored the existing knowledge about consumers behaviour of purchasing prescription medicines online. The prevalence of people purchasing prescription medicines from the Internet, as well as the factors that could influence consumers’ decisions to make this purchase, was highlighted in this review. Implications to policymakers were provided. Furthermore, research gaps were identified, and future research opportunities were discussed.

The behaviour of purchasing prescription medicines from the internet is complex as many factors could affect consumers decision to make the online purchase of prescription medicines. The current theory-based study helps resolve this complexity as this study explored those factors and provided an overarching understanding of the reasons that could encourage consumers to purchase medicines from the internet. Identifying those factors could enable the development of evidence-based public awareness campaigns to protect consumers from these purchase risks.

## Abbreviations

ASOP, Alliance for Safe Online Pharmacy; AXIS, Appraisal tool for Cross-Sectional Studies; CASP, Critical Appraisal Skills Programme; COM-B, the Capability, Opportunity, Motivation- Behaviour; GPhC, General Pharmaceutical Council; MHRA, Medicines and Healthcare products Regulatory Agency; MMAT, Mixed Methods Appraisal Tool; PRISMA, Preferred Reporting Items for Systematic reviews and Meta-Analyses; TDF, Theoretical Domains Framework.

## References

[B1] Abanmy N. (2017). The extent of use of online pharmacies in Saudi Arabia. Saudi Pharm. J. 25 (6), 891–899. 10.1016/j.jsps.2017.02.001 28951675 PMC5605957

[B2] Aiken A. R. Broussard K. Johnson D. M. Padron E. (2018). Motivations and experiences of people seeking medication abortion online in the United States. Perspect. Sex. reproductive health 50 (4), 157–163. 10.1363/psrh.12073 PMC8256438 29992793

[B3] Ajzen I. (2020). The theory of planned behavior: frequently asked questions. Hum. Behav. Emerg. Technol. 2 (4), 314–324. 10.1002/hbe2.195

[B4] Alhusein N. Scott J. Neale J. Chater A. Family H. (2021). Community pharmacists' views on providing a reproductive health service to women receiving opioid substitution treatment: a qualitative study using the TDF and COM-B. Explor. Res. Clin. Soc. Pharm. 4, 100071. 10.1016/j.rcsop.2021.100071 34870263 PMC8626316

[B5] Almomani H. Patel N. Donyai P. (2023b). News media coverage of the problem of purchasing fake prescription medicines on the internet: thematic analysis. JMIR Form. Res. 7, e45147. 10.2196/45147 36943354 PMC10131998

[B6] Almomani H. Patel N. Donyai P. (2023a). Reasons that lead people to end up buying fake medicines on the internet: qualitative interview study. JMIR Form. Res. 7 (1), e42887. 10.2196/42887 36795460 PMC9982721

[B7] Alwhaibi M. Asser W. M. Al Aloola N. A. Alsalem N. Almomen A. Alhawassi T. M. (2021). Evaluating the frequency, consumers’ motivation and perception of online medicinal, herbal, and health products purchase safety in Saudi Arabia. Saudi Pharm. J. 29 (2), 166–172. 10.1016/j.jsps.2020.12.017 33679178 PMC7910136

[B8] Ashames A. Bhandare R. AlAbdin S. Z. Alhalabi T. Jassem F. (2019). Public perception toward e-commerce of medicines and comparative pharmaceutical quality assessment study of two different products of furosemide tablets from community and illicit online pharmacies. J. Pharm. Bioallied Sci. 11 (3), 284–291. 10.4103/jpbs.JPBS_66_19 31555036 PMC6662035

[B9] ASOP (2020). 2020 National survey on American perception of online pharmacies. Alliance for Safe Online Pharmacy. Available at: https://asopfoundation.pharmacy/wp-content/uploads/2021/07/Survey-Key-Findings_October-2020.pdf .

[B10] ASOP (2022). The Alliance for Safe Online Pharmacies is dedicated to combatting illegal online pharmacies and counterfeit medicines to make the Internet safer for consumers worldwide. Alliance for Safe Online Pharmacy. Available at: https://buysaferx.pharmacy/ .

[B11] Assi S. Thomas J. Haffar M. Osselton D. (2016). Exploring consumer and patient knowledge, behavior, and attitude toward medicinal and lifestyle products purchased from the internet: a web-based survey. JMIR public health surveillance 2 (2), e34. 10.2196/publichealth.5390 27430264 PMC4969549

[B12] Atkins L. Francis J. Islam R. O’Connor D. Patey A. Ivers N. (2017). A guide to using the Theoretical Domains Framework of behaviour change to investigate implementation problems. Implement. Sci. 12 (1), 77–18. 10.1186/s13012-017-0605-9 28637486 PMC5480145

[B13] Badreldin H. A. Atallah B. (2021). Global drug shortages due to COVID-19: impact on patient care and mitigation strategies. Res. Soc. Adm. Pharm. 17 (1), 1946–1949. 10.1016/j.sapharm.2020.05.017 PMC7235598 32446652

[B14] Barnett-Page E. Thomas J. (2009). Methods for the synthesis of qualitative research: a critical review. BMC Med. Res. Methodol. 9 (1), 59–11. 10.1186/1471-2288-9-59 19671152 PMC3224695

[B15] Bell E. Bryman A. Harley B. (2022). Business research methods. United Kingdom: Oxford University Press.

[B16] Bourlakis M. Papagiannidis S. Fox H. (2008). E-Consumer behaviour: past, present and future trajectories of an evolving retail revolution. Int. J. E-Business Res. (IJEBR) 4 (3), 64–76. 10.4018/jebr.2008070104

[B17] Bowman C. Family H. Agius-Muscat H. Cordina M. Sutton J. (2020). Consumer internet purchasing of medicines using a population sample: a mixed methodology approach. Res. Soc. Adm. Pharm. 16 (6), 819–827. 10.1016/j.sapharm.2019.09.056 31668549

[B18] Boyd S. E. Moore L. S. P. Gilchrist M. Costelloe C. Castro-Sánchez E. Franklin B. D. (2017). Obtaining antibiotics online from within the UK: a cross-sectional study. J. Antimicrob. Chemother. 72 (5), 1521–1528. 10.1093/jac/dkx003 28333179 PMC5890662

[B19] Cane J. O’Connor D. Michie S. (2012). Validation of the theoretical domains framework for use in behaviour change and implementation research. Implement. Sci. 7 (1), 37–17. 10.1186/1748-5908-7-37 22530986 PMC3483008

[B20] CASP (2018). CASP qualitative checklist. Critical appraisal Skills Programme. Available at: https://casp-uk.net/wp-content/uploads/2018/01/CASP-Qualitative-Checklist-2018.pdf (Accessed December 20, 2022).

[B21] Cicero T. J. Ellis M. S. (2012). Health outcomes in patients using no-prescription online pharmacies to purchase prescription drugs. J. Med. Internet Res. 14 (6), e174. 10.2196/jmir.2236 23220405 PMC3799543

[B22] Demant J. Bakken S. A. Hall A. (2020). Social media markets for prescription drugs: platforms as virtual mortars for drug types and dealers. Drugs Alcohol Today 20 (1), 36–49. 10.1108/dat-06-2019-0026

[B23] Dobersek U. Wy G. Adkins J. Altmeyer S. Krout K. Lavie C. J. (2021). Meat and mental health: a systematic review of meat abstention and depression, anxiety, and related phenomena. Crit. Rev. Food Sci. Nutr. 61 (4), 622–635. 10.1080/10408398.2020.1741505 32308009

[B24] Downes M. J. Brennan M. L. Williams H. C. Dean R. S. (2016). Development of a critical appraisal tool to assess the quality of cross-sectional studies (AXIS). BMJ open 6 (12), e011458. 10.1136/bmjopen-2016-011458 PMC5168618 27932337

[B25] European Medicines Agency (2019). Falsified medicines: overview. Available at: https://www.ema.europa.eu/en/human-regulatory/overview/public-health-threats/falsified-medicines-overview .

[B26] Fittler A. Ambrus T. Serefko A. Smejkalová L. Kijewska A. Szopa A. (2022). Attitudes and behaviors regarding online pharmacies in the aftermath of COVID-19 pandemic: at the tipping point towards the new normal. Front. Pharmacol. 13, 1070473. 10.3389/fphar.2022.1070473 36642991 PMC9833114

[B27] Fittler A. Bősze G. Botz L. (2013a). Evaluating aspects of online medication safety in long-term follow-up of 136 internet pharmacies: illegal rogue online pharmacies flourish and are long-lived. J. Med. Internet Res. 15 (9), e199. 10.2196/jmir.2606 24021777 PMC3785996

[B28] Fittler A. Lankó E. Brachmann B. Botz L. (2013b). Behaviour analysis of patients who purchase medicines on the internet: can hospital pharmacists facilitate online medication safety? Eur. J. Hosp. Pharm. 20 (1), 8–12. 10.1136/ejhpharm-2012-000085

[B29] Fittler A. Vida R. G. Káplár M. Botz L. (2018a). Consumers turning to the internet pharmacy market: cross-sectional study on the frequency and attitudes of Hungarian patients purchasing medications online. J. Med. Internet Res. 20 (8), e11115. 10.2196/11115 30135053 PMC6125612

[B30] Fittler A. Vida R. G. Rádics V. Botz L. (2018b). A challenge for healthcare but just another opportunity for illegitimate online sellers: dubious market of shortage oncology drugs. PLoS One 13 (8), e0203185. 10.1371/journal.pone.0203185 30153304 PMC6112670

[B31] Gabay M. (2015). Regulation of internet pharmacies: a continuing challenge. Hosp. Pharm. 50 (8), 681–682. 10.1310/hpj5008-681 26823617 PMC4686473

[B32] GPC (2022). Guidance for registered pharmacies providing pharmacy services at a distance, including on the internet. General Pharmaceutical Council. Available at: https://www.pharmacyregulation.org/sites/default/files/document/guidance-for-registered-pharmacies-providing-pharmacy-services-at-a-distance-including-on-the-internet-march-2022.pdf .

[B33] Hafsteinsdóttir T. B. van der Zwaag A. M. Schuurmans M. J. (2017). Leadership mentoring in nursing research, career development and scholarly productivity: A systematic review. Int. J. Nurs. Stud. 75, 21–34. 10.1016/j.ijnurstu.2017.07.004 28710936

[B34] Haidich A. B. (2010). Meta-analysis in medical research. Hippokratia 14 (1), 29–37. 21487488 PMC3049418

[B35] Harden A. Garcia J. Oliver S. Rees R. Shepherd J. Brunton G. (2004). Applying systematic review methods to studies of people’s views: an example from public health research. J. Epidemiol. Community Health 58, 794–800. 10.1136/jech.2003.014829 15310807 PMC1732892

[B36] Hock S. Lee M. Chan L. (2019). Regulating online pharmacies and medicinal product E-commerce. United States: Pharmaceutical Engineering.

[B37] Hockenhull J. Wood D. M. Dargan P. I. (2020). The availability of modafinil and methylphenidate purchased from the internet in the United Kingdom without a prescription. Subst. Use Misuse 55 (1), 56–65. 10.1080/10826084.2019.1654516 31431114

[B38] Hong Q. N. Fàbregues S. Bartlett G. Boardman F. Cargo M. Dagenais P. (2018). The Mixed Methods Appraisal Tool (MMAT) version 2018 for information professionals and researchers. Educ. Inf. 34 (4), 285–291. 10.3233/efi-180221

[B39] Iyengar S. Hedman L. Forte G. Hill S. (2016). Medicine shortages: a commentary on causes and mitigation strategies. BMC Med. 14 (1), 124–133. 10.1186/s12916-016-0674-7 27683105 PMC5041339

[B40] Jairoun A. A. Al-Hemyari S. S. Abdulla N. M. El-Dahiyat F. Jairoun M. Al-Tamimi S. K. (2021). Online medication purchasing during the covid-19 pandemic: a pilot study from the United Arab Emirates. J. Pharm. Policy Pract. 14 (1), 38–47. 10.1186/s40545-021-00320-z 33931118 PMC8086226

[B41] Koenraadt R. Van de Ven K. (2018). The internet and lifestyle drugs: an analysis of demographic characteristics, methods, and motives of online purchasers of illicit lifestyle drugs in the Netherlands. Drugs 25 (4), 345–355. 10.1080/09687637.2017.1369936

[B42] Kulshrestha A. Mayer J. R. (2021). Identifying harmful media in end-to-end encrypted communication: efficient private membership computation. in USENIX security symposium (Germany: Springer), 893–910.

[B43] Lee K. S. Yee S. M. Zaidi S. T. R. Patel R. P. Yang Q. Al-Worafi Y. M. (2017). Combating sale of counterfeit and falsified medicines online: a losing battle. Front. Pharmacol. 8, 268. 10.3389/fphar.2017.00268 28559845 PMC5432535

[B44] Liang B. A. Mackey T. K. (2012). Vaccine shortages and suspect online pharmacy sellers. Vaccine 30 (2), 105–108. 10.1016/j.vaccine.2011.11.016 22094281

[B45] Little A. Sinclair M. Gillen P. Zheng H. (2018). Online medication purchasing behaviour in pregnancy: a structured review of the literature. Evid. Based Midwifery 16 (1), 13–20.

[B46] Little A. Sinclair M. Zheng H. Mackin P. (2020). Factors that influence online medication purchasing behaviour in pregnancy: a qualitative study. Evid. Based Midwifery 30 (2), 159–171.

[B47] Long C. S. Kumaran H. Goh K. W. Bakrin F. S. Ming L. C. Rehman I. U. (2022). Online pharmacies selling prescription drugs: systematic review. Pharmacy 10 (2), 42. 10.3390/pharmacy10020042 35448701 PMC9031186

[B48] Lundin S. Liu R. (2020). Where and how do you buy medicines? A pilot survey of consumption strategies among the public in Sweden. J. Public Health 42 (3), e268–e271. 10.1093/pubmed/fdz075 PMC7435214 31334767

[B49] Mackey T. K. Nayyar G. (2016). Digital danger: a review of the global public health, patient safety and cybersecurity threats posed by illicit online pharmacies. Br. Med. Bull. 118 (1), 110–126. 10.1093/bmb/ldw016 27151957 PMC5127424

[B50] MHRA (2022). Protect your health when buying medicines online. Fakemeds. Available at: https://fakemeds.campaign.gov.uk/ .

[B51] Michie S. Johnston M. Abraham C. Lawton R. Parker D. Walker A. (2005). Making psychological theory useful for implementing evidence based practice: a consensus approach. BMJ Qual. Saf. 14 (1), 26–33. 10.1136/qshc.2004.011155 PMC1743963 15692000

[B52] Michie S. Van Stralen M. M. West R. (2011). The behaviour change wheel: a new method for characterising and designing behaviour change interventions. Implement. Sci. 6 (1), 42–12. 10.1186/1748-5908-6-42 21513547 PMC3096582

[B53] Monteith S. Glenn T. (2018). Searching online to buy commonly prescribed psychiatric drugs. Psychiatry Res. 260, 248–254. 10.1016/j.psychres.2017.11.037 29220682

[B54] Moor L. Anderson J. R. (2019). A systematic literature review of the relationship between dark personality traits and antisocial online behaviours. Personality Individ. Differ. 144, 40–55. 10.1016/j.paid.2019.02.027

[B55] Moureaud C. Hertig J. Dong Y. Muraro I. S. Alhabash S. (2021). Purchase of prescription medicines via social media: A survey-based study of prevalence, risk perceptions, and motivations. Health Policy 125 (11), 1421–1429. 10.1016/j.healthpol.2021.09.007 34625280

[B56] Musazzi U. M. Di Giorgio D. Minghetti P. (2020). New regulatory strategies to manage medicines shortages in Europe. Int. J. Pharm. 579, 119171. 10.1016/j.ijpharm.2020.119171 32092455 PMC7125892

[B57] OECD/EUIPO (2020). Trade in counterfeit pharmaceutical products, illicit trade. Paris: OECD Publishing.

[B58] Orizio G. Merla A. Schulz P. J. Gelatti U. (2011). Quality of online pharmacies and websites selling prescription drugs: a systematic review. J. Med. Internet Res. 13 (3), e74. 10.2196/jmir.1795 21965220 PMC3222188

[B59] Page M. J. McKenzie J. E. Bossuyt P. M. Boutron I. Hoffmann T. C. Mulrow C. D. (2021). The PRISMA 2020 statement: an updated guideline for reporting systematic reviews. Syst. Rev. 10 (1), 790–799. 10.1016/j.rec.2021.07.010 PMC8008539 33781348

[B60] Pál S. Laszlo K. Fittler A. Hancu G. Finta H. Ciurba A. (2015). Attitude of patients and customers regarding purchasing drugs online. Farmacia 63 (1), 93–98.

[B61] Pavlou P. A. Fygenson M. (2006). Understanding and predicting electronic commerce adoption: an extension of the theory of planned behavior. MIS Q. 30, 115–143. 10.2307/25148720

[B62] Pavlou P. A. (2002). “What drive electronic commerce? A theory of planned behaviour perspective,” in Academy of management proceedings (Briarcliff Manor, NY: Academy of Management), A1–A6.

[B63] Reeves S. Albert M. Kuper A. Hodges B. D. (2008). Why use theories in qualitative research? Bmj 337, a949. 10.1136/bmj.a949 18687730

[B64] Reichert A. Jacobs R. (2018). The impact of waiting time on patient outcomes: evidence from early intervention in psychosis services in england. Health Econ. 27 (11), 1772–1787. 10.1002/hec.3800 30014544 PMC6221005

[B65] Sun N. Gong Y. Liu J. Wu J. An R. Dong Y. (2021). Prevalence of antibiotic purchase online and associated factors among Chinese residents: a nationwide community survey of 2019. Front. Pharmacol. 12, 761086. 10.3389/fphar.2021.761086 34803704 PMC8595837

[B66] Svorc J. (2012). Consumer's intentions to shop medicaments on-line: a survey from Czech republic market. J. Syst. Integration 3 (2), 3.

[B67] Taherdoost H. (2022). What are different research approaches? Comprehensive review of qualitative, quantitative, and mixed method research, their applications, types, and limitations. J. Manag. Sci. Eng. Res. 5 (1), 53–63. 10.30564/jmser.v5i1.4538

[B68] Thomas J. Harden A. (2008). Methods for the thematic synthesis of qualitative research in systematic reviews. BMC Med. Res. Methodol. 8 (1), 45–10. 10.1186/1471-2288-8-45 18616818 PMC2478656

[B69] Timlin D. McCormack J. M. Simpson E. E. (2021). Using the COM-B model to identify barriers and facilitators towards adoption of a diet associated with cognitive function (MIND diet). Public Health Nutr. 24 (7), 1657–1670. 10.1017/S1368980020001445 32799963 PMC8094434

[B70] West R. Michie S. (2020). A brief introduction to the COM-B Model of behaviour and the PRIME Theory of motivation [v1]. Article WW04E6. Available at: https://discovery.ucl.ac.uk/id/eprint/10095640 .

[B71] Whitfield M. Germain J. Hillis A. Halsall D. McVeigh J. Abbasi Y. (2021). Internet sourcing and UK end consumer trend interest in the controlled medicines (opioids, sedatives and GABA drugs) in pre and post COVID-19 timeframes. Emerg. Trends Drugs, Addict. Health 1, 100027. 10.1016/j.etdah.2021.100027

[B72] Yaylı A. Bayram M. (2012). E-WOM: the effects of online consumer reviews on purchasing decisions. Int. J. Internet Mark. Advert. 7 (1), 51–64. 10.1504/IJIMA.2012.044958

[B73] Zou H. Chen Y. Fang W. Zhang Y. Fan X. (2017). Identification of factors associated with self-care behaviors using the COM-B model in patients with chronic heart failure. Eur. J. Cardiovasc. Nurs. 16 (6), 530–538. 10.1177/1474515117695722 28756696

