# Supplementary material for: Reasons that lead people to buy prescription medicines on the internet: a systematic review
Source: Front Pharmacol. 2023 Aug 31;14:1239507. doi: 10.3389/fphar.2023.1239507 (PMC10501782; doi:10.3389/fphar.2023.1239507)
Supplement: Supplementary file 1 [file Table1.DOCX]

Table 1. The Theoretical Domains Framework domains and definitions [Cane et al., 2012 and Michie et al., 2005 as cited in Lavalléea et al., 2018]

| TDF-14 domains  *(Source: Cane et al., 2012)* | Definitions |
| --- | --- |
| Knowledge | The knowledge of the existence of something, the risks associated with it, and the procedural knowledge. |
| Skills | Cognitive or physical abilities acquired via practice. |
| Social/professional role and identity | Professional identity, social identity, identity, professional confidence, or group identify. |
| Beliefs about capabilities | Self-confident, perceived competence, self-efficacy, perceived behavioural control beliefs, self-esteem, empowerment, professional confidence. |
| Beliefs about consequences | Positive or negative outcomes of a specific behaviour. |
| Goals | Target that an individual wants to achieve. |
| Memory, Attention and Decision Processes | The ability to retain information |
| Environmental Context and Resources | Any environmental condition that discourages or encourages the development of skills, abilities, or adaptive behaviour |
| Social influences | Social influencing factors including social pressure, social norms, social comparisons, or groups norms. |
| Emotion | A complex reaction pattern by which people attempt to deal with a personally significant event. |
| Behavioural Regulation | Anything aimed at managing or controlling specific actions (e.g., facilitators and barriers). |
| Optimism | The high level of confidence that things will happen for the good. |
| Reinforcement | Rewards, incentives, punishment, or reinforcement. |
| Intentions | A conscious decision to perform a behaviour. |
