# Supplementary material for: Reasons that lead people to buy prescription medicines on the internet: a systematic review
Source: Front Pharmacol. 2023 Aug 31;14:1239507. doi: 10.3389/fphar.2023.1239507 (PMC10501782; doi:10.3389/fphar.2023.1239507)
Supplement: Supplementary file 2 [file Table2.DOCX]

**CASP Qualitative Studies Checklist**

Note: This tool developed for a critical assessment of the quality of qualitative studies

Answers: [Yes = 1] [No or Can’t Tell (CT) = 0]

| **Questions** | **1QL** | **2QL** | **3QL** | **4QL** |
| --- | --- | --- | --- | --- |
| **Section A: Are the results valid?** | | | | |
| 1. Was there a clear statement of the aims of the research? | Y | Y | Y | Y |
| 1. Is a qualitative methodology appropriate? | Y | Y | Y | Y |
| **Is it worth continuing?** | | | | |
| 1. Was the research design appropriate to address the aims of the research? | Y | Y | Y | Y |
| 1. Was the recruitment strategy appropriate to the aims of the research? | Y | Y | Y | Y |
| 1. Was the data collected in a way that addressed the research issue? | Y | Y | Y | Y |
| 1. Has the relationship between researcher and participants been adequately considered? | Y | CT | CT | CT |
| **Section B: What are the results?** | | | | |
| 1. Have ethical issues been taken into consideration? | Y | Y | CT | Y |
| 1. Was the data analysis sufficiently rigorous? | N | CT | CT | Y |
| 1. Is there a clear statement of findings? | Y | Y | Y | Y |
| **Section C: Will the results help locally?** | | | | |
| 1. Is the research valuable? | Y | Y | Y | Y |
| **Score** | **9** | **8** | **7** | **9** |
