# Supplementary material for: Reasons that lead people to buy prescription medicines on the internet: a systematic review
Source: Front Pharmacol. 2023 Aug 31;14:1239507. doi: 10.3389/fphar.2023.1239507 (PMC10501782; doi:10.3389/fphar.2023.1239507)
Supplement: Supplementary file 3 [file Table5.docx]

Quantitative Part Findings

# **Quantitative studies and the quantitative part of the mixed method study’s findings**

| Code | Citation | Analysis used | Type of medicines purchased | Factors that could influence people decision to buy medicines online |
| --- | --- | --- | --- | --- |
| 1QN | Alwhaibi *et al.,* 2021 | Descriptive analysis (frequencies and percentages) and chi square | POMs + OTC   - Viagra - Birth control bills - Antibiotic - Narcotics - Refill medication for chronic condition - Herbal medicine, supplements. - Cosmetics | **Motivational factors for online purchasing of medicinal products:**   - Lower cost. - Variety of discount offers. - Convenience - easy access to medicines information from home at any time. - Convenience - it reduces visits to health care professionals and community pharmacies. - Availability of wide variety of medicines. - Privacy. - Accessibility. - Availability of detailed information about the products. - Better quality products. - Unsatisfied with the quality of clinical services provided in local community pharmacy. - Unavailability of medicines in the local market.   **What are the risks of purchasing medicinal products from the internet?**   - It could be unsafe purchase. - The difficulty in distinguishing between registered online pharmacies and other unlicensed commercial websites. - Instructions in an unknown foreign language. - Product quality concerns. - Lack of supervision of a healthcare professional. - No proper information regarding the use of the products. - People may get medicinal products that they don’t need that can worsen their condition.   **Where did participants hear about online medicine purchasing?**   - Social media/internet websites. - Family. - Friends or co-workers. - Health care provider (physician, pharmacist, nurse).   **Demographics:**   - Age. - Gender. - Education. - Employment. - Income. |
| 2QN | Jairoun et al., 2021 | Descriptive + inferential statistics (CI) | POMs and OTC medicines   - Dietary supplements - Analgesics - Antihistamines - Anti-cough medicine | **Motivational factor:**   - COVID-19 outbreak.   **Socio-demographics characteristics:**   - Sex. - Marital status. - Education. - Age. |
| 3QN | Moureaud *et al.,* 2021 | Descriptive statistics (Mean and SD) + Inferential statistics (binomial logistic regression) | POMs   - Sedatives (Xanax®, Valium®, Ativan®, etc.). - Stimulants (Adderall®, Ritalin®, etc.). - Narcotics (Vicodin®, Percocet®, Oxycontin®, fentanyl, etc.). - COVID-19 medicines or vaccines. | **Motivational factor:**   - Getting legitimate medicine. - low price. - Purchasing from a source recommended by people I know. - Being able to purchase from a reliable source. - Getting a generic prescription medicine. - Getting a brand name prescription medicine. - Being able to purchase in bulk. - Being able to purchase without a prescription. - Being able to purchase non-FDA approved treatment.   **Demographic characteristics:**   - The higher a participant’s education, the lower the odds for purchasing each type of medicines online. - Employment was associated with increased odds of buying different types of medicines online. - Social media affinity (favourable attitudes toward social media) positively predicted the odds of purchasing prescription medicines online.   **Risk perception:**   - The higher risk perceived by the participants, the lower the probability of purchasing online.   **Past purchase behaviour**   - Past experience with purchasing medicines on online pharmacies, e-retailer sites, and instant messaging services, respectively, predicted purchase behaviour. |
| 4QN | Ashames *et al.,* 2019 | Descriptive analysis (frequencies and percentages) | POMs + OTC | **Motivational factors:**   - Low prices. - The nonavailability of certain medications in local Pharmacies. - No prescription needed. - Wide choice of products. - Not sure about the quality of the purchased online medication. - Unaware about the law of UAE that considers purchasing medications from online sources as illegal. - Believed in high quality of online medications.   **Demographics:**   - Education. - Age. |
| 5QN | Fittler *et al.,* 2018a | Descriptive statistics (Mean and SD) + correlation (for demographics) | POMs + OTC | **Motivational factors:**   - Convenient. - People who cannot get to a pharmacy can also purchase products. - People can purchase medicines after opening hours. - People can access products which are otherwise not available. - Fast. - Products can be compared faster and more easily than in the pharmacy. - Inexpensive. - Can get more information compared to the pharmacy. - Can get products with better quality compared to the pharmacy.   **Potential disadvantages:**   - It is easier to abuse preparations. - People can get products they do not need or worsen their condition. - I do not get proper information regarding the use of the products. - Due to the delivery time, I'm getting the drug later compared to a pharmacy. - The source of the product is not reliable. - It is hard for me to choose between the great numbers of products. - Possibility of not getting the right product. - Possibility of getting counterfeit medicine. - The quality of the product is lower compared than in local pharmacies.   **Demographics and internet using habits that could influence the purchasing decision:**   - Level of education (People possessing higher level college degrees and having a more positive attitude regarding online medication purchase, are more likely to do so). - The younger generation is much more involved in the online market. - Internet purchase frequency in general. - Average time spent on the internet |
| 6QN | Koenraadt & Van de Ven, 2018 | Descriptive statistics (Mean and SD) | POMs | **Motivational factors:**   - Convenience. - Low prices. - Home delivery. - Helpful if consumers do not want to make a discussion with the doctor. - Able to order after office hours. - Advised by others. - Helpful if consumers cannot receive a prescription. - Can obtain products that are not for sale in Netherlands. - More effective.   **Trust factors:**   - Clear information about vendors and medicines. - Clear overview of available lifestyle drugs. - Reviews by others. - Vendor s known. - Vendor’s expertise. - Satisfaction. |
| 7QN | Abanmy, 2017 | Descriptive analysis (frequencies and percentages) | POMs + OTC | **Reasons to buying medicines online:**   - Unavailable in local market. - Cheaper. - More convenient. - Good services such as home delivery and refill reminder by email. - Available 24 h, 7 day a week. - Providing health information and some consultation. - Easy delivery especially for those far from any community pharmacy. - More privacy. - Avoid bad services of community pharmacy such as long waiting time. - Satisfaction with the online seller.   **Reasons for not buying medicines online:**   - No license. - Quality of the medicine. - Simple prescription. - Extra money. - No privacy and confidentiality. - No idea about online pharmacy. - Medicines needed is available in community pharmacy. - No trust. - No interest. - Medicines available in hospitals. - Bad mail services. - Inappropriate storage. - Not allowed to enter Saudi Arabia.   **Demographics:**   - Age. - Gender. - Education. - Income. |
| 8QN | Assi *et al.,* 2016 | Descriptive analysis (frequencies and percentages) | POMs + OTC | **Reasons for buying products online:**   - Quick/time saving. - Cheap. - Easy. - Convenient. - More details on product than ones provided by the pharmacist. - No need for embarrassment of communicating with pharmacist. - Lack of availability of products in stores. - Not able to get prescription for some products. - Wide variety of products and offers. - Ability to purchase illegal drugs. - Better quality products. - Medicine shortage. |
| 9QN | Szekely *et al.,* 2015 | Descriptive analysis (frequencies and percentages)  Pearson Chi-Square was used to compare demographic variable effect on the purchasing behaviour. | POMs +OTC | **Factors that could influence the purchasing decision:**   - Quality concerns (some believed medicine available online are with inferior quality while others’ beliefs it is come with a better quality. - Better quality. - Cheaper. - Awareness about the hazardous effect of medicines purchased online (safety concerns). - Still consider that information from pharmacists and physicians is necessary to start using a new medicine with confidence. (Lack of medical oversight). - One-third of the subjects consider that the information from healthcare professionals is not by all means necessary, and they can get sufficient information about a new medicine from the medicine leaflet, the Internet or from marketing flyers.   **Safety evaluation for drugs purchased from the Internet:**   - Significantly more men than women think that it is not unsafe to purchase medicines online. |
| 10QN | Fittler *et al.,* 2013b | Descriptive analysis (frequencies and percentages) to calculate variables and (mean and SD) for age. | POMs + OTC | **Motivational factors:**   - Lower prices. - Low awareness about the risks associated with purchasing medicines online. - Online advertisements. - Websites containing information on medicines. - Time spends on the internet. |
| 11QN | Cicero & Ellis, 2012 | Chi-square and logistical regression analyses at a P<.01 level. | Tramadol | **Motivational factors:**   - Accessibility (cannot find doctor who will prescribe, doctor will not prescribe enough, no other way to get it, convenience). - Economic (cheaper, Lack of healthcare coverage, do not want to pay for doctor). - Others (Anonymity, curiosity, prevent withdrawals, hate going to doctor, others-not specified).   **Demographics and health information:**   - Age.   Health care coverage. |
| 12QN | Svorc, 2012 | Factor analysis, t-test, multiple regression analysis. | POMs + OTC | **Motivation factors:**   - Consumer perceived usefulness. - Consumer perceived ease of use. - Subjective norms (celebrities and people who consumers care about). - Past experience with online shopping. - Low perceived risk of online shopping. - Trust. |
| Code | **Citation** | **Analysis used** | **Type of medicines purchased** | **Factors that could influence people decision to buy medicines online** |
| 1MX | Bowman et al., 2020 | Descriptive statistics. Chi-square tests were used to establish associations between demographics and various responses. | POMs + OTC | **Motivational factors:**   - Had experienced using prescription-only medicines (POM) for themselves. - Cheaper. - Medicine is not found locally. - More convenient that going to local pharmacy. - Saw advert through the internet. - Brand availability. - Advised by friend or family member. - Not entitled to get them for free through the national health service (NHS) (medicines that are not covered by the insurance). - Medicines are not available from the NHS (Medicines not available locally). - Side effect from previous purchase. - Safety concerns. - Trusted source. - Quality concerns. - Satisfaction level with healthcare services.   **Demographics:**   - Age. - Educational level. |
