# Supplementary material for: Reasons that lead people to buy prescription medicines on the internet: a systematic review
Source: Front Pharmacol. 2023 Aug 31;14:1239507. doi: 10.3389/fphar.2023.1239507 (PMC10501782; doi:10.3389/fphar.2023.1239507)
Supplement: Supplementary file 4 [file Table3.DOCX]

**Mixed Methods Appraisal Tool (MATT) (2018 version)**

Note: This tool developed for a critical assessment of the quality of mixed methods studies

Answers: [Yes = 1] [No or Can’t Tell (CT) = 0]

| **Questions** | **1 MX** | |
| --- | --- | --- |
| **Screening questions** | |  |
| 1. Are there clear research questions? | Y |  |
| 1. Are there clear research questions? | Y |  |
| **Qualitative part** | |  |
| 1. Is the qualitative approach appropriate to answer the research question? | Y |  |
| 1. Are the qualitative data collection methods adequate to address the research question? | Y |  |
| 1. Are the findings adequately derived from the data? | Y |  |
| 1. Is the interpretation of results sufficiently substantiated by data? | Y |  |
| 1. Is there coherence between qualitative data sources, collection, analysis, and interpretation? | Y |  |
| **Quantitative part** | |  |
| 1. Is the sampling strategy relevant to address the research question? | Y |  |
| 1. Is the sample representative of the target population? | Y |  |
| 1. Are the measurements appropriate? | Y |  |
| 1. Is the risk of nonresponse bias low? | CT |  |
| 1. Is the statistical analysis appropriate to answer the research question? | Y |  |
| **Mixed Methods part** | |  |
| 1. Is there an adequate rationale for using a mixed methods design to address the research question? | Y |  |
| 1. Are the different components of the study effectively integrated to answer the research question? | Y |  |
| 1. Are the outputs of the integration of qualitative and quantitative components adequately interpreted? | Y |  |
| 1. Are divergences and inconsistencies between quantitative and qualitative results adequately addressed? | Y |  |
| 1. Do the different components of the study adhere to the quality criteria of each tradition of the methods involved? | Y |  |
| **Score** | **16** |  |
