# Supplementary material for: Reasons that lead people to buy prescription medicines on the internet: a systematic review
Source: Front Pharmacol. 2023 Aug 31;14:1239507. doi: 10.3389/fphar.2023.1239507 (PMC10501782; doi:10.3389/fphar.2023.1239507)
Supplement: Supplementary file 5 [file Table4.DOCX]

**AXIS quality assessment checklist**

Note: This tool developed for a critical assessment of the quality of cross-sectional studies

Answers: Yes / No / Do not know (DK)

Yes = 1, No and DK = 0

| **Questions** | **1 QN** | **2 QN** | **3 QN** | **4 QN** | **5 QN** | **6 QN** | **7 QN** | **8 QN** | **9 QN** | **10 QN** | **11 QN** | **12 QN** |
| --- | --- | --- | --- | --- | --- | --- | --- | --- | --- | --- | --- | --- |
| **Introduction** | | | | | | | | | | | | |
| 1. Were the aims/objectives of the study clear? | Y | Y | Y | Y | Y | Y | Y | Y | Y | Y | Y | Y |
| **Methods** | | | | | | | | | | | | |
| 1. Was the study design appropriate for the stated aim(s)? | Y | Y | Y | Y | Y | Y | Y | Y | Y | Y | Y | Y |
| 1. Was the sample size justified? | Y | N | N | Y | N | N | N | N | N | N | N | N |
| 1. Was the target/reference population clearly defined? (Is it clear who the research was about?) | Y | Y | Y | Y | Y | Y | Y | Y | Y | Y | Y | Y |
| 1. Was the sample frame taken from an appropriate population base so that it closely represented the target population under investigation? | Y | Y | Y | Y | Y | Y | Y | Y | Y | Y | Y | N |
| 1. Was the selection process likely to select participants that were representative of the target population under investigation? | DK | DK | Y | DK | Y | Y | Y | Y | DK | DK | DK | DK |
| 1. Were measures undertaken to address non-responders? | DK | DK | DK | DK | N | DK | DK | N | N | DK | DK | N |
| 1. Were the outcome variables measured appropriate to the aims of the study? | Y | Y | Y | Y | Y | Y | Y | Y | Y | Y | Y | Y |
| 1. Were the outcome variables measured correctly using instruments/ measurements that had been trialled, piloted, or published previously? | Y | DK | DK | DK | Y | Y | Y | Y | DK | DK | DK | Y |
| 1. Is it clear what was used to determined statistical significance and/or precision estimates? (e.g., p values, CIs) | N | Y | N | N | Y | N | N | N | Y | Y | N | Y |
| 1. Were the methods (including statistical methods) sufficiently described to enable them to be repeated? | Y | Y | Y | Y | Y | Y | Y | Y | Y | Y | Y | Y |
| **Results** | | | | | | | | | | | | |
| 1. Were the basic data adequately described? | Y | Y | Y | Y | Y | Y | Y | Y | Y | Y | Y | Y |
| 1. Does the response rate raise concerns about nonresponse bias? | DK | DK | DK | Y | N | Y | N | DK | DK | N | DK | N |
| 1. If appropriate, was information about non-responders described? | N | N | Y | N | N | N | N | N | N | N | N | N |
| 1. Were the results internally consistent? | Y | Y | Y | Y | Y | Y | Y | Y | Y | Y | Y | Y |
| 1. Were the results for the analyses described in the methods, presented? | Y | Y | Y | Y | Y | Y | Y | Y | Y | Y | Y | Y |
| **Discussion** | | | | | | | | | | | | |
| 1. Were the authors’ discussions and conclusions justified by the results? | Y | Y | Y | Y | Y | Y | Y | Y | Y | Y | Y | Y |
| 1. Were the limitations of the study discussed? | Y | Y | Y | N | Y | Y | Y | Y | Y | Y | Y | Y |
| **Others** | | | | | | | | | | | | |
| 1. Were there any funding sources or conflicts of interest that may affect the authors’ interpretation of the results? | Y | N | N | Y | N | Y | Y | N | Y | Y | N | DK |
| 1. Was ethical approval or consent of participants attained? | Y | Y | Y | Y | Y | Y | Y | Y | Y | Y | N | Y |
| Score (out of 20) | **15** | **13** | **14** | **14** | **15** | **16** | **15** | **14** | **14** | **14** | **11** | **13** |
